# Aortic systolic and pulse pressure invasively and non-invasively obtained: Comparative analysis of recording techniques, arterial sites of measurement, waveform analysis algorithms and calibration methods

**DOI:** 10.3389/fphys.2023.1113972

**Published:** 2023-01-16

**Authors:** Daniel Bia, Yanina Zócalo, Ramiro Sánchez, Gustavo Lev, Oscar Mendiz, Franco Pessana, Agustín Ramirez, Edmundo I. Cabrera-Fischer

**Affiliations:** ^1^ Departamento de Fisiología, Centro Universitario de Investigación, Innovación y Diagnóstico Arterial (CUiiDARTE), Facultad de Medicina, Universidad de la República, Montevideo, Uruguay; ^2^ Metabolic Unit and Hypertension Unit, University Hospital, Favaloro Foundation, Buenos Aires, Argentina; ^3^ Department of Interventional Cardiology, University Hospital, Favaloro Foundation, Buenos Aires, Argentina; ^4^ Department of Information Technology, Engineering and Exact Sciences Faculty, Favaloro University, Buenos Aires, Argentina; ^5^ IMETTYB Favaloro University—CONICET, Buenos Aires, Argentina

**Keywords:** applanation tonometry, calibration, catheterism, central aortic blood pressure, invasive records, non-invasive records, oscillometry, vascular ultrasound

## Abstract

**Background:** The non-invasive estimation of aortic systolic (aoSBP) and pulse pressure (aoPP) is achieved by a great variety of devices, which differ markedly in the: 1) principles of recording (applied technology), 2) arterial recording site, 3) model and mathematical analysis applied to signals, and/or 4) calibration scheme. The most reliable non-invasive procedure to obtain aoSBP and aoPP is not well established.

**Aim:** To evaluate the agreement between aoSBP and aoPP values invasively and non-invasively obtained using different: 1) recording techniques (tonometry, oscilometry/plethysmography, ultrasound), 2) recording sites [radial, brachial (BA) and carotid artery (CCA)], 3) waveform analysis algorithms (e.g., direct analysis of the CCA pulse waveform vs. peripheral waveform analysis using general transfer functions, N-point moving average filters, etc.), 4) calibration schemes (systolic-diastolic calibration vs. methods using BA diastolic and mean blood pressure (bMBP); the latter calculated using different equations vs. measured directly by oscillometry, and 5) different equations to estimate bMBP (i.e., using a form factor of 33% (“033”), 41.2% (“0412”) or 33% corrected for heart rate (“033HR”).

**Methods:** The invasive aortic (aoBP) and brachial pressure (bBP) (catheterization), and the non-invasive aoBP and bBP were simultaneously obtained in 34 subjects. Non-invasive aoBP levels were obtained using different techniques, analysis methods, recording sites, and calibration schemes.

**Results:** 1) Overall, non-invasive approaches yielded lower aoSBP and aoPP levels than those recorded invasively. 2) aoSBP and aoPP determinations based on CCA recordings, followed by BA recordings, were those that yielded values closest to those recorded invasively. 3) The “033HR” and “0412” calibration schemes ensured the lowest mean error, and the “033” method determined aoBP levels furthest from those recorded invasively. 4) Most of the non-invasive approaches considered overestimated and underestimated aoSBP at low (i.e., 80 mmHg) and high (i.e., 180 mmHg) invasive aoSBP values, respectively. 5) The higher the invasively measured aoPP, the higher the level of underestimation provided by the non-invasive methods.

**Conclusion:** The recording method and site, the mathematical method/model used to quantify aoSBP and aoPP, and to calibrate waveforms, are essential when estimating aoBP. Our study strongly emphasizes the need for methodological transparency and consensus for the non-invasive aoBP assessment.

## 1 Introduction

The non-invasive estimation of aortic blood pressure (aoBP) is done using a variety of commercial devices that differ: 1) in the principles considered for recording the pulse waveform or surrogate signals (applied technology), 2) in the arterial site of recording, and/or 3) in the model and mathematical analysis applied (Papaioannou et al., 2016-A; Chi et al., 2022; Jedrzejewski et al., 2021). Most devices use plethysmography, applanation tonometry or vascular ultrasound to obtain radial (RA), brachial (BA) or common carotid (CCA) arteries’ pulse waveforms. Then, from the obtained waveforms, and after their calibration, the devices quantify aortic systolic and pulse pressure (aoSBP, aoPP) “directly” (e.g., direct calibration of CCA waveforms) or “indirectly,” for instance, applying generalized transfer functions (GTF), low-pass filters (e.g., N-point moving average, NPMA) or wave analysis algorithms (e.g., detection of the second shoulder in the radial wave) (Papaioannou et al., 2016-A; Chi et al., 2022). Several studies have been developed to assess the validity of specific approaches and commercial devices used to determine central aortic pressure. In this context, it is worth mentioning that up to now, there is no consensus on which (if any) would be the best device/technique, site of recording and/or mathematical algorithm to be used (Papaioannou et al., 2016-A). In this regard, differences in the applied technology, device models and/or algorithms of waveform analysis used could result in differences in aoSBP and aoPP levels (Papaioannou et al., 2016-A). On the other hand, when the devices are calibrated using non-invasive peripheral blood pressure (BP) (e.g., non-invasive brachial BP, bBP), the agreement between invasive and non-invasive aoBP data would be “calibration mode-dependent” and significant differences between invasive and non-invasive aoBP levels could be observed. Furthermore, it is currently accepted that the main drawback (major source of error) when determining aoBP (aoSBP or aoPP) is the method of calibration used (Hope et al., 2004; Papaioannou et al., 2006; Nakagomi et al., 2017; Weber et al., 2011; Wassertheurer et al., 2018; Negishi et al., 2016; Papaioannou et al., 2016-A). Related with this, it is to note that the calibration scheme that minimizes error when using a specific device and/or methodology may not be the same when using another approach.

In both, physiological research and clinical practice two bBP-associated calibration schemes have been mostly used: 1) calibration to systolic (bSBP) and diastolic (bDBP) BP (termed systo-diastolic calibration [SD]), and 2) calibration to bDBP and mean blood pressure (bMBP) (Diaz et al., 2021; Diaz et al., 2019; Papaioannou et al., 2016-A). The bMBP levels to be used for calibration and aoBP assessment could correspond to bMBP 1) *measured* by oscillometry (OscM), obtained from the lowest cuff pressure value measured during the maximum oscillations’ plateau or 2) *calculated* (CM) from bSBP and bDBP, using different scaling forms (Papaioannou et al., 2016-A). In this context, it remains to be analyzed whether the differences between invasive and non-invasive aoSBP (or aoPP) levels obtained with the same or different devices or algorithms would be (significantly) modified by the calibration scheme and/or the approach used to determine bMBP. An additional issue to be considered is the impact on the obtained data of the way in which bMBP is determined from bSBP and bDBP. About this, equations that differ in the form factor (FF) (e.g., 33% [0.33], 41.2% [0.412]), in the use of mathematical corrections by heart rate (HR) have been proposed and used to obtain bMBP (Mahiu et al., 2010; Laugesen et al., 2014). Papaioannou et al. (2016) reported that compared with the traditional formula that uses a FF of 0.33, the use of a FF equal to 0.412 would be superior to discriminate subjects with left ventricular and carotid wall hypertrophy, as well as with increased aortic stiffness. However, it remains to be seen whether using a FF of 0.412 (rather than the proposed 0.33 or other bMBP-related equation), reduces the differences between aoSBP (or aoPP) measured invasively and non-invasively, regardless of the device and/or algorithm used in pulse waveform analysis. Related with the above, at least in theory, as was mentioned by Wassertheurer et al. regarding to the best calibration scheme, “*results may be device-dependent*.” In this regard, data about the “best calibration scheme” come from studies that used the same device and it remains to be determined if findings could be extrapolated (and/or generalized) to other approaches (Sharman et al., 2017). Related with this, up to now there is no agreement on which should be considered the most reliable non-invasive procedure to obtain aoSBP and aoPP. In addition, taking into account the above, it is difficult to analyze, comparatively, results from studies that considered different devices/techniques, arterial recording sites, mathematical approaches, bMBP estimation methods, and/or calibration schemes (Agnoletti et al., 2012).

In this context, the aim of this study was to evaluate the association and agreement between aoSBP and aoPP values invasively and non-invasively obtained considering or using different:

i) recording techniques [applanation tonometry (SphygmoCor device), oscilometry/plethysmography (Mobil-O-Graph device), vascular ultrasound (SonoSite device)],
ii) recording sites (RA, BA and CCA),
iii) waveform analysis algorithms (e.g., direct analysis of the CCA pulse waveform or peripheral waveform analysis using GTF, NPMA, second radial shoulder, etc.,)
iv) calibration approaches (SD vs. OscM vs. CM), and
v) equations to estimate bMBP.

## 2 Methods

### 2.1 Subjects

Thirty-four subjects (41% females; 14–89 years-old) with coordinated coronary angiogram at the Favaloro Foundation University Hospital were included (Sánchez et al., 2020). Subjects with valvular heart disease and/or arrhythmia were excluded. A clinical interview, together with an anthropometric evaluation, enabled to assess the exposure to cardiovascular risk factors (CRFs), defined according to criteria previously described (Zócalo and Bia, 2021-A; Zócalo and Bia, 2021-B; Zócalo and Bia, 2022; Zócalo et al., 2021; Bia and Zócalo, 2021; Díaz et al., 2018). Laboratory biochemical data were obtained and echocardiographic examinations were performed (Table 1). Written informed consent was obtained from the participants and/or their parents. The study protocol was approved by the Institutional Ethic Committee. All procedures were performed in agreement with the Declaration of Helsinki.

**TABLE 1:** Demographic, anthropometric and clinical characteristics of the study population.

Variable	MV	SE	SD	Min	p25th	p50th	p75th	Max	Range
Age [years]	61	3	19	14	52	68	72	89	75
Body weight [kg]	75.5	2.6	15.3	46	65	73	88	103	57
Body height [m]	166	2	9	147	162	165	174	182	35
BMI [kg/m^2^]	27.2	0.8	4.4	17.5	24.2	27.0	29.9	38.9	21.3
Hemoglobin [g/l]	12.5	1.2	2.6	8.4	12.3	12.8	13.9	15.3	6.9
Hematocrit [%]	38.1	1.8	6.6	26.0	36.0	38.3	43.0	46.0	20.0
Total cholesterol [mg/dL]	184.0	22.5	59.5	122.0	131.0	170.0	239.0	287.0	165.0
HDL cholesterol [mg/dL]	45.7	5.1	13.5	29.0	39.0	41.0	54.0	71.0	42.0
LDL cholesterol [mg/dL]	114.7	23.7	62.6	64.0	66.0	78.0	186.0	218.0	154.0
Triglycerides [mg/dL]	119.1	22.8	60.2	69.0	71.0	103.0	151.0	238.0	169.0
Atherogenic index	4.45	0.89	2.36	2.56	2.85	3.05	7.36	8.24	5.68
Creatinine [mg/dL]	1.15	0.10	0.36	0.79	0.80	1.07	1.46	1.90	1.11
Urea [mg/dL]	50.0	6.6	22.9	28.0	33.0	38.5	66.0	99.0	71.0
Glycaemia [mg/dL]	109.4	14.0	41.9	74.0	89.0	90.0	106.0	197.0	123.0
Sodium [mEq/L]	132.7	1.5	4.6	122.0	132.0	133.0	136.0	137.0	15.0
Potassium [mEq/L]	4.1	0.2	0.5	3.4	3.7	4.3	4.6	4.7	1.3
LVEDD [mm]	52.3	2.9	10.4	38.0	42.0	53.0	59.0	71.0	33.0
LVESD [mm]	31.2	2.5	8.8	18.0	25.0	30.5	37.5	45.0	27.0
LV Septum thickness [mm]	10.5	0.7	2.4	6.8	8.0	11.6	12.0	14.0	7.2
LV Posterior wall thickness [mm]	9.2	0.6	2.0	6.5	7.0	9.0	11.0	12.0	5.5
Left atrium area [cm^2^]	26.5	2.1	5.1	18.0	25.0	26.5	30.0	33.0	15.0
LV Ejection fraction [%]	59	2	8	38	55	60	65	70	32
Active smokers [%]	5.9								
Ex-smokers [%]	48.3								
Arterial hypertension [%]	69.7								
Diabetes [%]	30.3								
Diabetics requiring insulin [%]	25.0								
Dyslipidemia [%]	60.6								
Renal insufficiency [%]	18.2								
Myocardial infarction [%]	18.2								
Acute coronary syndrome [%]	7.4								
CABG [%]	12.1								
Coronary Angioplasty [%]	15.2								
ACEI [%]	37.5								
ARBs [%]	29.2								
MRAs [%]	12.5								
Beta blockers [%]	50.0								
Diuretics [%]	20.8								
Calcium channel blockers [%]	29.2								
Antiplatelet therapy [%]	31.3								
Statins [%]	66.7								
T4 [%]	8.3								

ACEI, angiotensin-converting enzyme inhibitors; ARBs, angiotensin II, receptor blockers; BMI, body mass index; CABG, coronary artery bypass graft surgery; LV, left ventricle; EDD, and ESD, end-diastolic and end-systolic diameter; Min. and Max, minimal and maximal value; MRAs, Mineralocorticoid receptor antagonists; MV, mean value; SE, standard error of the mean; SD, standard deviation; p25th, p50th, and p75th, 25th (first quartile), 50th (median) and 75th percentile (third quartile).

**FIGURE 1:**
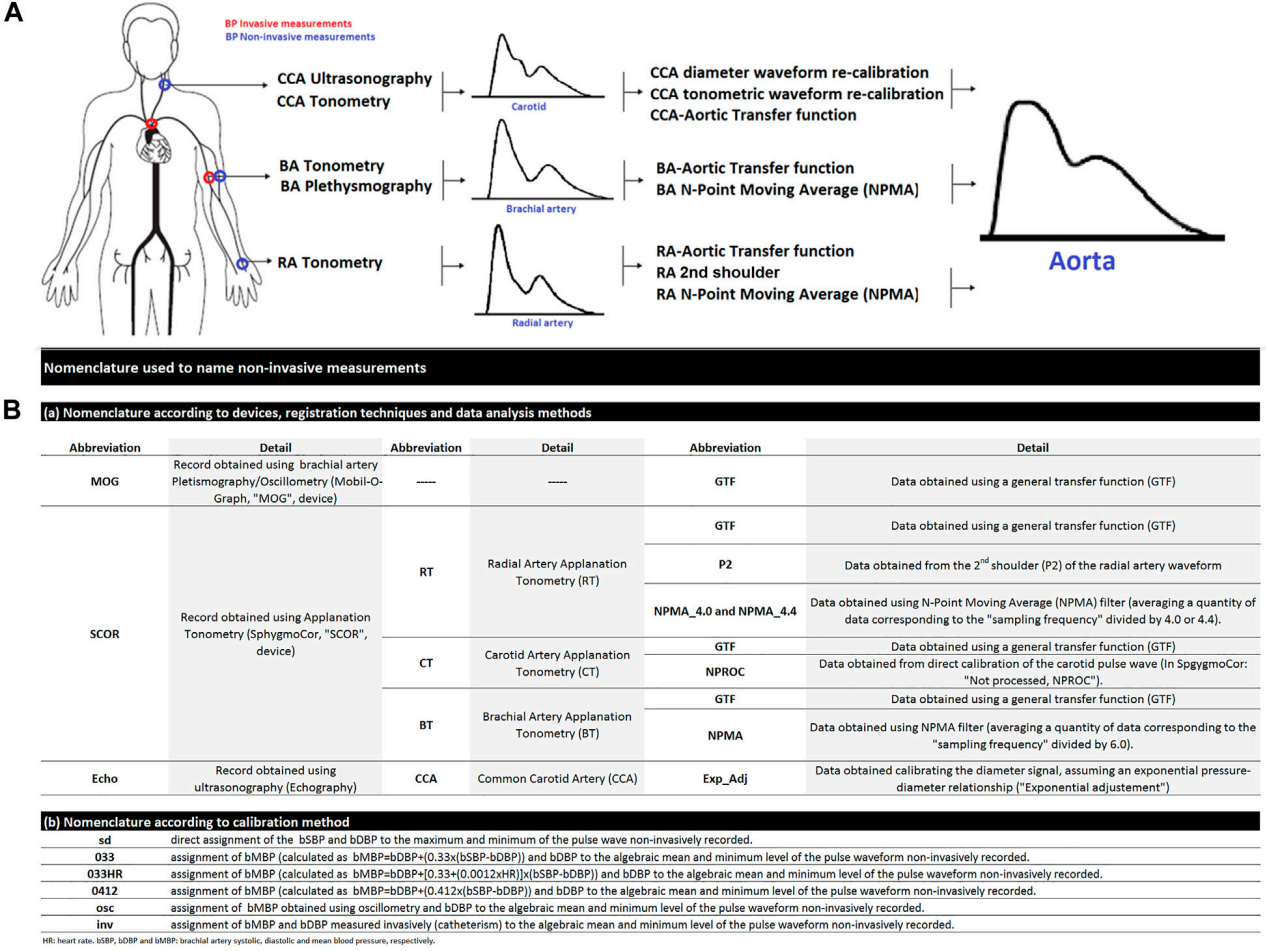
**(A)**: Schematic representation of invasive and non-invasive blood pressure (BP) measurements in the aortic root and brachial artery (A). CCA, common carotid artery. BA, brachial artery. RA, radial artery. In the **(B)**: nomenclature used to designate non-invasive derived variables.

The following recordings were performed in each subject: 1) invasive (catheterization) measurement of aoBP and bBP levels and waveforms, 2) non-invasive assessment of aoBP levels and waveforms using: 1) applanation tonometry (SphygmoCor device; SphygmoCor-CvMS ([SCOR]; v.9, AtCor-Medical, Sydney, NSW, Australia) recordings of the CCA, BA and RA level, 2) oscillometric/plethysmographic (Mobil-O-Graph device, Model PWA, IEM GmbH, Stolberg, Germany) recordings from the BA, and 3) vascular ultrasound (SonoSite device) recordings of the CCA (Figure 1).

### 2.2 Invasive measurement of central aortic and brachial artery blood pressure

Intra-arterial aoBP and bBP levels and waveforms were obtained with the subjects lying in the supine position. Briefly, standard asepsis was performed in the arterial access area (radial) followed by cutaneous/subcutaneous injection of lidocaine. A soft sedation was also administered as needed. After local anesthesia was applied in the vessel access area, a 5 or 6 French introducer sheath was positioned in the arterial lumen and heparin was administered through the arterial catheter. Subsequently, a 0.035-inch guide wire was placed in the ascending aorta, and a 5 French pig tail catheter (Cordis, Miami, United States) was introduced thereafter. Special attention was paid to place the catheter tip ∼4 cm away from the aortic valve. After confirming the correct positioning of the catheter (fluoroscopy), the guide wire was removed and the catheter was flushed with saline solution.

To record intravascular pressure, the aforementioned fluid-filled catheter placed in the proximal ascending aorta (or BA) was connected to the external transducer (MX960, Medex, LogiCal, Smiths Medical ASD Inc., Minneapolis, United States) (Billiet, 2007), and the transducer was connected to the AcistCVi system (AcistCVi, Medical System Inc., Germany). The AsistCVi system was synchronized with the X-ray imaging system Allura Xper FD10 or AlluraClarity FD20/10 (Philips Healthcare, the Netherlands).

Prior to each measurement, the combined system of catheter, tubing and external transducer was flushed with saline solution and the aoBP (or bBP) trace was visually inspected for quality. The external BP transducer was calibrated following the system’s inbuilt 2-point calibration method. The external transducer was always maintained at heart (mid-axillary line) level. Invasive BP waveforms were visualized in the Allura Xper FD10 or AlluraClarity FD20/10 monitor images (Philips Healthcare, the Netherlands).

Simultaneously with invasively obtained aoBP recordings, aoBP estimates were obtained using three approaches: ultrasound, oscillometry/plethysmography, and tonometry [see below] (Figure 1).

Once invasive aoBP recordings were obtained, the catheter was positioned in the contralateral BA at the level where the cuff for non-invasive aoBP and bBP measurement (Mobil-O-Graph) was located (Figure 1). Thereafter, intra-arterial bBP levels and waveforms were recorded and non-invasive bBP data were obtained (immediately before or after) using oscillometry (Mobil-O-Graph) [see below]. Following each invasive bBP recording, the catheter was again positioned in the aorta and aoBP levels and waveforms were recorded, allowing hemodynamic stability to be assessed (confirmed).

Systolic, diastolic, mean (i.e., area under the pressure/time curve, divided by the cycle length) BP levels and HR were determined by means of invasive-derived data analysis processing systems. Once invasive and non-invasive measurement procedures concluded, the catheter was removed, and each subject returned to the recovery area.

### 2.3 Non-invasive measurement of brachial blood pressure and determination of mean arterial blood pressure

bBP pressure and waveforms were recorded by oscillometry/plethysmography (Mobil-O-Graph), simultaneously and/or immediately before or after each invasive recording (at aortic or BA level). Non-invasive bBP values were used to calibrate pulse waveforms recorded at CCA (ultrasound), BA (tonometry, oscillometry/plethysmography), and RA (tonometry) level [see below].

The bSBP, bDBP, brachial pulse pressure (bPP = bSBP-bDBP), and HR values obtained with the oscillometric system (Mobil-O-Graph) were named bSBPosc, bDBPosc, bPPosc, and HRosc, respectively. The bMBP obtained with oscillometry (the point of lower bBP for maximal oscillations) was identified as bMBPosc. Additionally, using the bSBPosc and bDBPosc, bMBP was quantified in three different ways (Papaioannou et al., 2016-B; Agnoletti et al., 2012; Chemla et al., 2005):

i) bMBP_0.33_ [mmHg] = bDBPosc+0.33*bPPosc
ii) bMBP_0.33HR_ [mmHg] = bDBPosc+[0.33+(0.0012*HRosc)]*bPPosc
iii) bMBP_0.412_ [mmHg] = bDBPosc+0.412*bPPosc

These approaches use two different “form factors” (33% [0.33] and 41.2% [0.412]), defined as the percentage of the waveform amplitude (the bPP) that is added to the minimum (the bDBPosc) to obtain the mean value (the bMBP) (Mahieu et al., 2010). These different ways of obtaining bMBP were subsequently used to calibrate the ultrasound, tonometry, and oscillometry/plethysmography recordings used to obtain aoBP, with the aim of determining whether any of these equations allow for smaller differences between aoSBP (and aoPP) obtained invasively and non-invasively.

### 2.4 Non-invasive measurement of central aortic blood pressure

Simultaneously with invasive aoBP recordings, aoBP estimates were obtained (random order) using different non-invasive approaches, whose recording techniques and mathematical algorithms are detailed below.

#### 2.4.1 aoBP estimated from brachial oscillometry/plethysmography recordings

Oscillometric/plethysmographic bBP levels and waveforms recordings were obtained with the Mobil-O-Graph device. To this end, a pneumatic cuff was positioned in the arm (in our case in the contralateral to the used for the sheath insertion) (Figure 1) (Weber et al., 2011; García-Espinosa et al., 2016; Zinoveev et al., 2019; Zócalo and Bia, 2022). Then, HRosc and bMBPosc were registered, and bSBPosc and bDBPosc were obtained, by means of internal algorithms of the device manufacturer. In turn, aoBP levels and waveforms were estimated from BA recordings using a validated GTF. Only high-quality records (index equal to 1 or 2) and waveforms (visual inspection) were considered.

The Mobil-O-Graph device determines bBP and aoBP during the same “double” inflation-deflation cycle of the cuff. This device was used: 1) to determine aoBP, and 2) to determine the bSBPosc, bMBPosc, and bDBPosc values used in its own calibration and in the calibration of other approaches. Therefore, every time bBP was measured with this device, along with the measurement with another non-invasive approach (e.g., tonometry, ultrasound), aoBP was also determined using this device.

Each aoBP data derived from Mobil-O-Graph recordings was obtained calibrating to: 1) bSBPosc and bDBPosc (SD approach); 2) bDBPosc and bMBPosc, and 3) bDBPosc and bMBP_0.33._ It was not possible to calibrate Mobil-O-Graph derived data using invasive bBP levels (catheterism-derived) or other forms of estimating bMBP (i.e., bMBP_0.33HR_, bMBP_0.412_) or), as the device does not allow it.

#### 2.4.2 aoBP estimated from carotid ultrasonography recordings

Left CCAs were visualized a centimeter proximal to the bulb using ultrasound (6–13 MHz, M-Turbo, Sonosite Inc, Bothell, WA, United States). Sequences of images (30 s, B-Mode, longitudinal views) were stored for off-line analysis in which beat-to-beat diameter waveforms were obtained using border detection software (Hemoydin4M software, Dinap s.r.l., Buenos Aires, Argentina). Then, aoBP waveform and values were obtained from the diameter data (Van Bortel et al., 2001; Vermeersch et al., 2008; Zócalo et al., 2013). CCA diameter waveforms were calibrated applying the method proposed by Vermeersch et al. that assumes an exponential arterial pressure-diameter relationship (Vermeersch et al., 2008; Zócalo et al., 2013). To use this equation to calculate the BP waveform from a given diameter waveform, systolic and diastolic BP must be known at the same site as the arterial cross section (diameter). Assuming that (in supine position) DBP and MBP remain constant throughout large arteries, 1) invasive-derived bDBP and bMBP, and 2) bDBPosc and bMBP levels were used to calibrate CCA diameter waveforms. Specifically, CCA ultrasound-derived aoSBP and aoPP were obtained using four different calibration schemes that included bDBPosc in conjunction with: 1) bMBPosc, and bMBPcalc [2) bMBP_0.33_, 3) bMBP_0.33HR_, 4) bMBP_0.412_].

#### 2.4.3 aoBP estimated from carotid applanation tonometry recordings

aoBP levels and waveforms were obtained (random order) using tonometry (SphygmoCor) applied to CCA, RA, and BA (Figure 1). Applanation tonometry provides the beat-to-beat BP waveform signal (10 s) that can be calibrated using different schemes. Only accurate waveforms on visual inspection and high-quality recordings (in-device quality control index >75%) were considered.

From the CCA tonometry-derived recordings, aoBP levels were obtained: 1) applying a carotid-aortic GTF (“GTF approach”) and 2) without using a GTF (not-processed or “NPROC” approach’), considering CCA and ascending aorta waveforms to be identical due to the proximity of the arterial sites (Karamanoglu and Feneley, 1996; Chi et al., 2022).

Disregard of the approach considered to obtain aoBP levels from CCA tonometry-derived data (as well as from RA and BA data as described below) signals were calibrated to: 1) invasively-derived bDBP and bMBP, and non-invasively derived, 2) bSBPosc and bDBPosc, and 3) bDBPosc and bMBP, using different ways to quantify bMBP: bMBPosc, bMBP_0.33_, bMBP_0.33HR_, bMBP_0.412_.

#### 2.4.4 aoBP estimated from radial applanation tonometry recordings

aoBP levels and/or waveforms were obtained from RA tonometry-derived pulse waveforms, considering different data analysis approaches.

First, the aoBP waveform, aoSBP, and aoPP levels were obtained applying a radial-to-aortic GTF (manufacturer’s property) (Castro et al., 2016; Zinoveev et al., 2019; Zócalo and Bia, 2022).

Second, aoSBP and aoPP were quantified from the second peak of the RA pulse waveform (usually referred to as “second shoulder, P2 or SBP2”) (Pauca et al., 2004; Hickson et al., 2009; Protogerou et al., 2010).

Third, aoSBP and aoPP were quantified applying a first-order low-pass filter: “N-point moving average” (NPMA) method. Each single point in the recorded signal (e.g., RA waveform) is summed up with its neighbors and the result is divided by the number of points considered. The more data points are taken into the average formula, the smoother the signal. The NPMA method was proposed as a simple method to estimate aoSBP from both, RA-derived (Williams et al., 2011; Weber et al., 2014) or BA-derived BP waveforms (Shih et al., 2014). The number of averaged points (“N”) differs depending on the BP waveforms (RA or BA) considered. For instance, N=Fs/4 (Williams et al., 2011) or N=Fs/4.4 (Xiao et al., 2018) for RA-derived waves, and N=Fs/6 for waves obtained from BA recordings (Shih et al., 2014) (Fs is the sampling frequency, and the numbers 4.0, 4.4 and 6 represent the optimal integer denominator “K”). Recently Xiao et al. (2018) reported that K = 4.4 would estimate aoSBP more accurately than K = 4.0. With this in mind, in this work we applied the NPMA method using both denominators (4.0 and 4.4) when analyzing RA-tonometry recordings. Using the SphygmoCor device (Fs = 128 Hz), 32 (128/4), and 29 (128/4.4) points were averaged for RA waveforms, while 21 (128/6) points were considered when analyzing BA records (see below).

#### 2.4.5 aoBP estimated from brachial applanation tonometry recordings

From tonometry-derived BA pulse waveforms (calibrated using different methods), we quantified aoBP: 1) applying the SphygmoCor GTF, and 2) using NPMA method (k = 6) (Shih et al., 2014).

### 2.5 Carotid, radial and brachial artery waveforms calibration

All devices that allow non-invasive acquisition of the pulse waveforms require a calibration step, in order to transform voltage (e.g., mV) or diameter (e.g., mm) signals, into BP (e.g., mmHg) waveforms. Different approaches were considered to calibrate the peripheral waveforms:

1) Invasive-derived (“Inv”): bMBP and bDBP invasively obtained (catheterization) were respectively assigned, to the algebraic mean and minimum of the CCA, RA, or BA waveforms non-invasively recorded.
2) Systo-diastolic (“SD”): bSBPosc and bDBPosc were respectively assigned to the maximum and minimum of the CCA, BA or RA waveforms non-invasively recorded.
3) Oscillometric-derived (“Osc”): bMBPosc and bDBPosc were respectively assigned to the algebraic mean and minimum of the CCA, RA or BA waveforms non-invasively recorded.
4) Calculated MBP: Calculated bMBP (bMBP_033_, bMBP_033HR_, bMBP_0412_) and bDBPosc were respectively assigned to the algebraic mean and minimum of the CCA, RA or BA waveforms non-invasively recorded.

### 2.6 Variable names: aoSBP and aoPP

The variables were named as a sequence of terms (separated by underscores) referring to (Figure 1):

i) The biological variable analyzed: aoSBP or aoPP
ii) The recording technique/device used: “MOG” (Mobil-O-Graph: Oscillometry/plethysmography); “SCOR” (SphygmoCor: Applanation tonometry); “Echo” (Echography or vascular ultrasound),
iii) The arterial site of recording: “CT” or “CCA” (carotid tonometry or carotid ultrasound, respectively), “BT” (brachial artery tonometry), “RT” (radial artery tonometry),
iv) The analysis considered: “GTF” (use of a general transfer function), “NPMA” (use of the N-point moving average filter; indicating the filtering factor used in RA: ‘4.0′ or “4.4,” and in BA: “6.0”), “ExpAdj” (use of an exponential fit for the diameter-pressure transformation),
v) The calibration form of the recorded waveforms: “Inv,” “sd,” “osc,” or the specific equation (form factor) used to calibrate using the calculated bMBP (“033,” “033HR,” “0412”).

As an example, the variable “aoSBP (MOG_GTF_sd)” refers to the aortic systolic blood pressure (“aoSBP”) measured with the Mobil-O-Graph (“MOG”), using a generalized transfer function (“GTF”) and obtained when calibrating using the systo-diastolic scheme (“sd”).

An extended version of this section (“*Methods*”) can be found in Supplementary File S1.

### 2.7 Data and statistical analysis

Invasive and non-invasive bBP and aoBP data obtained with the different techniques, recording sites, data analyses and/or calibration schemes are shown in the Supplementary File S2, Tables S1–S5 show: 1) aortic and BA data invasively obtained, 2) bBP values used to calibrate the signals (obtained simultaneously with the Mobil-O-Graph) and 3) aoBP data obtained from the non-invasive approaches: Supplementary Table S1: Oscilometry/Plethismography (Mobil-O-Graph), Supplementary Table S2: RA tonometry (SCOR), Supplementary Table S3: CCA tonometry (SCOR), Supplementary Table S4: BA tonometry (SCOR), Supplementary Table S5: CCA ultrasound (SonoSite + HemoDyn 4M-software).

**FIGURE 2:**
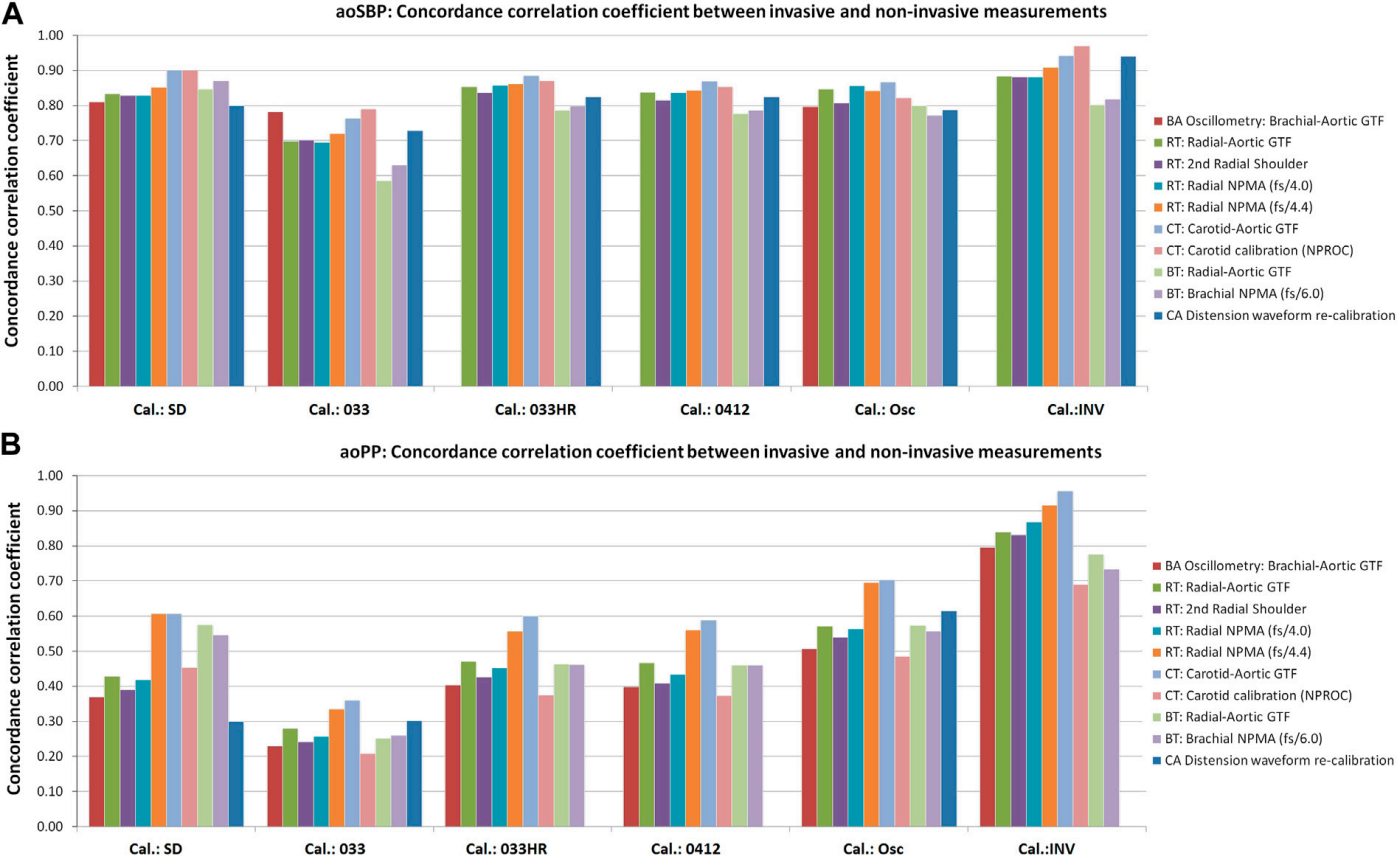
Concordance correlation coefficients (CCC) between invasive and non-invasive aoSBP **(A)** and aoPP **(B)** measurements. Detailed 95% confidence intervals for the CCC values are presented inSupplementary File S2 (Tables S6, S7).

**FIGURE 3:**
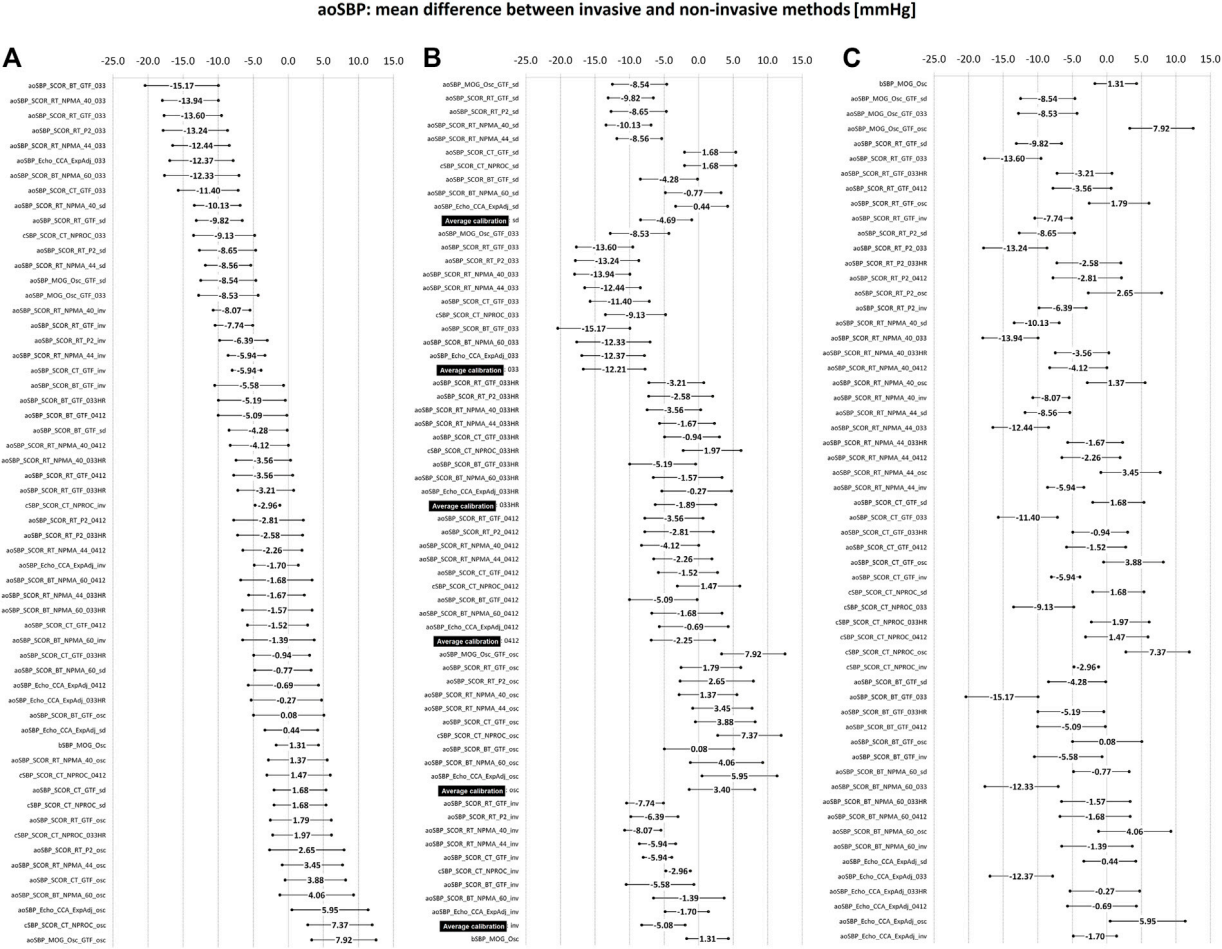
Bland-Altman derived mean error (and 95% confidence intervals) for comparisons between non-invasive and invasive aoSBP measurements. **(A)**: ordered according to error level. **(B)**: sorted by calibration scheme. **(C)**: sorted by methodology of obtaining the aoSBP value. Detailed quantitative information on the Bland-Altman test is provided in Supplementary File S2 (Table S8).

**FIGURE 4:**
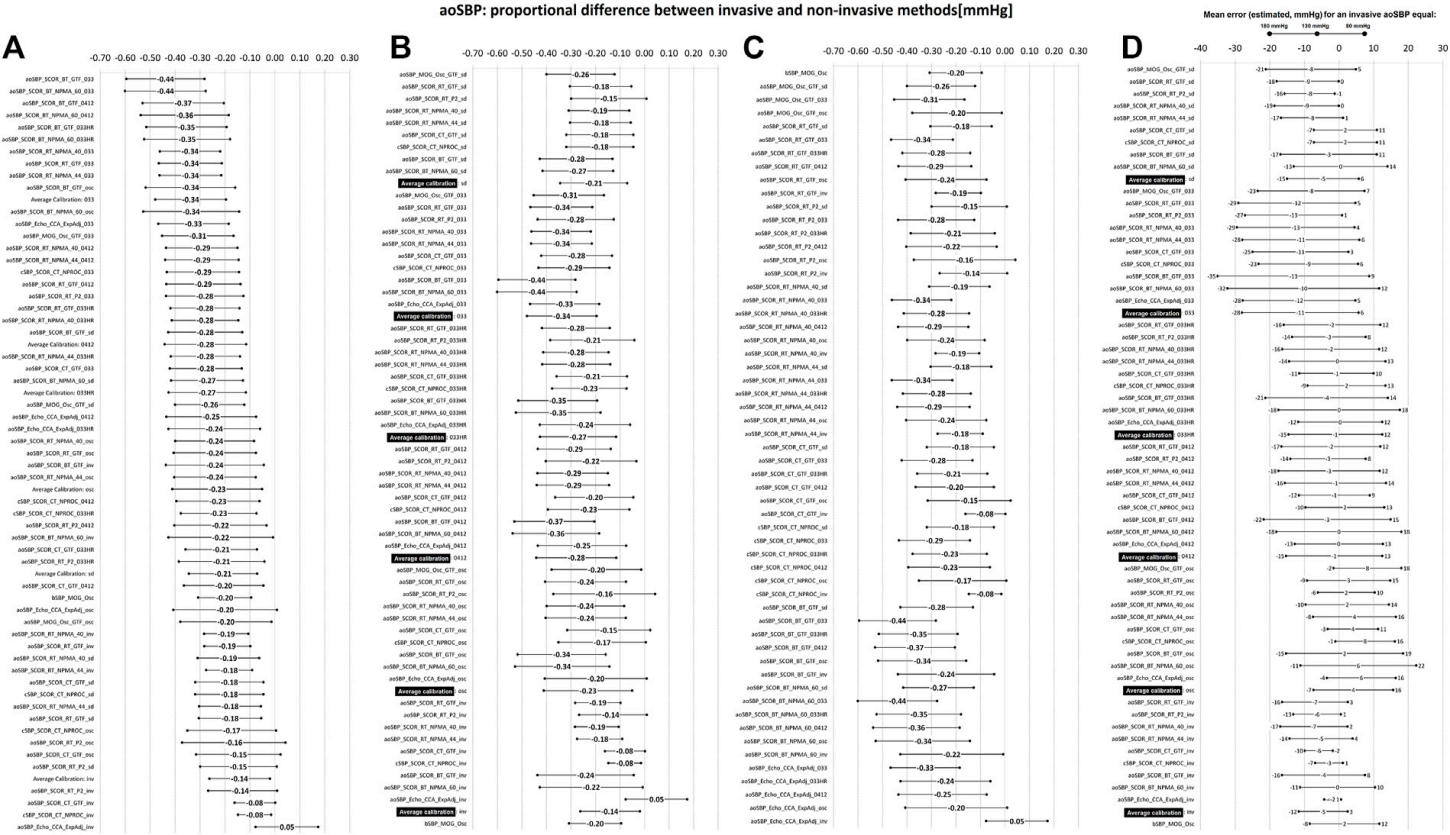
Bland-Altman derived proportional error (and 95% confidence interval) for comparisons between non-invasive and invasive aoSBP measurements. **(A)**: ordered according to error level. **(B)**: sorted by calibration scheme. **(C)**: sorted according to method used to obtain aoSBP. **(D)**: error (in mmHg) between non-invasive and invasive method plotted for three different invasive aoSBP levels: 80, 130 and 180 mmHg. Detailed quantitative information on Bland-Altman test is provided in Supplementary File S2 (Table S8).

**FIGURE 5:**
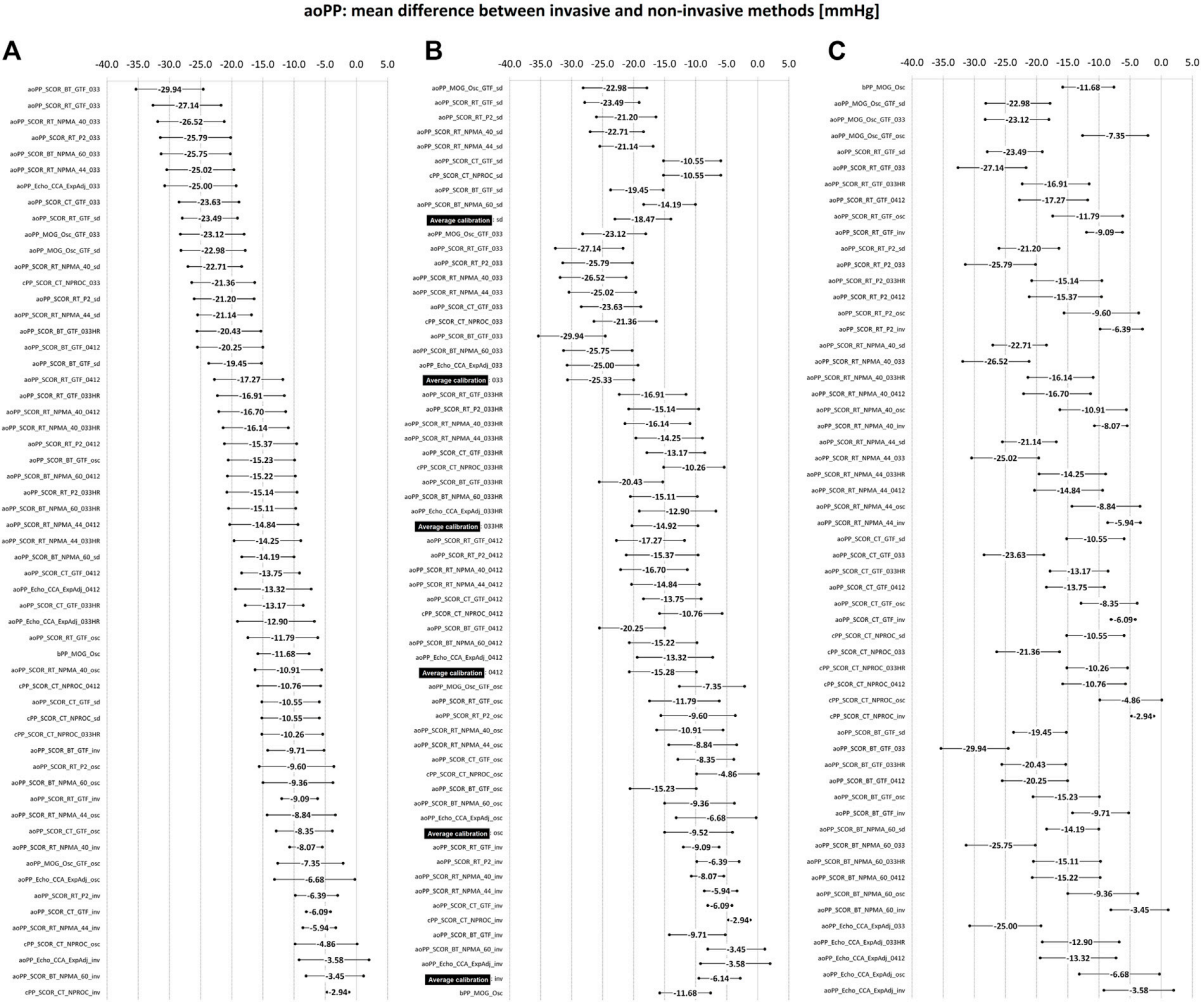
Bland-Altman derived mean error (and 95% confidence interval) for comparisons between non-invasive and invasive aoPP measurements. **(A)**: ordered according to the level of error. **(B)**: sorted by calibration scheme. **(C)**: sorted by methodology for obtaining the aoPP value. Detailed quantitative information on the Bland-Altman test is presented in Supplementary File S2 (Table S9).

**FIGURE 6:**
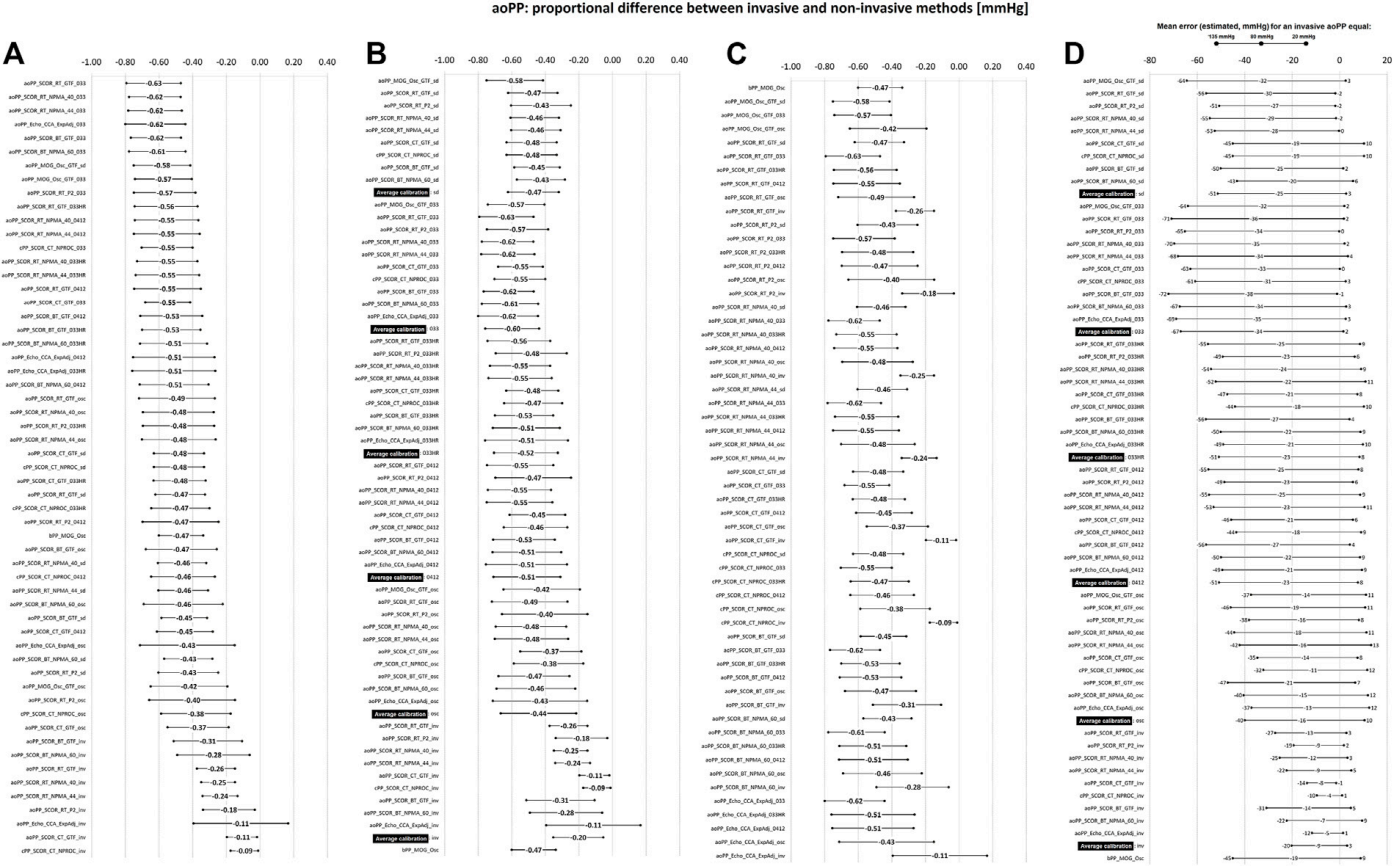
Bland-Altman derived proportional error (and 95% confidence interval) for comparisons between non-invasive and invasive aoPP measurements. **(A)**: ordered according to error level. **(B)**: ordered according to calibration scheme. **(C)**: ordered according to method used to obtaining aoPP value. **(D)**: error (in mmHg) between non-invasive and invasive methods at three different levels of invasive aoPP: 20, 80, and 135 mmHg. Detailed quantitative information about Bland-Altman test was reported in Supplementary File S2 (Table S9).

After analyzing the subjects’ characteristics, aoBP and bBP data obtained with the different approaches (Tables 1, 2; Supplementary File S2, Tables S1–S5), we analyzed the association and agreement between invasive and non-invasive aoSBP and aoPP data. To this end, Lin’s Concordance Correlation Coefficient (CCC) [Figure 2; Supplementary File S2, Table S6 (for aoSBP) and Supplementary Table S7 (for aoPP)], and Bland-Altman test were considered (Supplementary File S2, Tables S8, S9). Bland-Altman test enabled to determine mean (systematic) and proportional errors (bias) between aoSBP (Figures 3, 4) and aoPP (Figures 5, 6) data obtained with the reference (invasive) method and the non-invasive ones.

**TABLE 2:** Invasive aortic, and invasive and non-invasive (oscillometry) brachial pressure levels.

Variable	MV	SE	SD	Min	p25th	p50th	p75th	Max	Range
Invasive bSBP [mmHg]	146	5	28	77	133	144	168	189	112
Invasive bMBP [mmHg]	98	3	15	66	89	98	111	122	57
Invasive bDBP [mmHg]	71	2	10	54	63	70	80	92	38
Invasive bPP [mmHg]	75	5	25	21	57	75	92	135	114
Invasive aoSBP [mmHg]	135	4	23	77	122	134	154	179	102
Invasive aoMBP [mmHg]	94	3	14	65	85	91	104	121	56
Invasive aoDBP [mmHg]	68	2	10	52	62	65	75	92	40
Invasive aoPP [mmHg]	67	4	20	22	55	64	82	103	81
Invasive HR [beat/minute]	70	3	14	49	56	68	78	104	55
bSBP (Osc) [mmHg]	137	3	19	85	127	135	156	167	82
bDBP (Osc) [mmHg]	81	2	13	55	73	79	90	108	53
bPP (Osc) [mmHg]	56	3	14	29	45	55	67	91	62
HR (Osc) [mmHg]	71	3	14	44	59	72	81	105	61
bMBP_0.412_ [mmHg]	104	2	14	68	96	106	111	130	62
bMBP_0.33_ [mmHg]	100	2	13	65	92	100	106	125	60
bMBP_0.33HR_ [mmHg]	104	3	14	68	96	104	110	131	63
bMBP_osc_ [mmHg]	107	3	14	69	98	108	115	133	64

Prefix “b” and “ao” indicate brachial artery and aorta, respectively. HR, heart rate; MV, mean value; SBP, MBP, DBP, and PP, systolic, mean, diastolic and pulse blood pressure, respectively; SD, standard deviation; SE, standard error of the mean; p25th, p50th and p75th, 25th percentile (first quartile), 50th percentile (median) and 75th percentile (third quartile); Osc, oscillometry/plethysmography.

**FIGURE 7:**
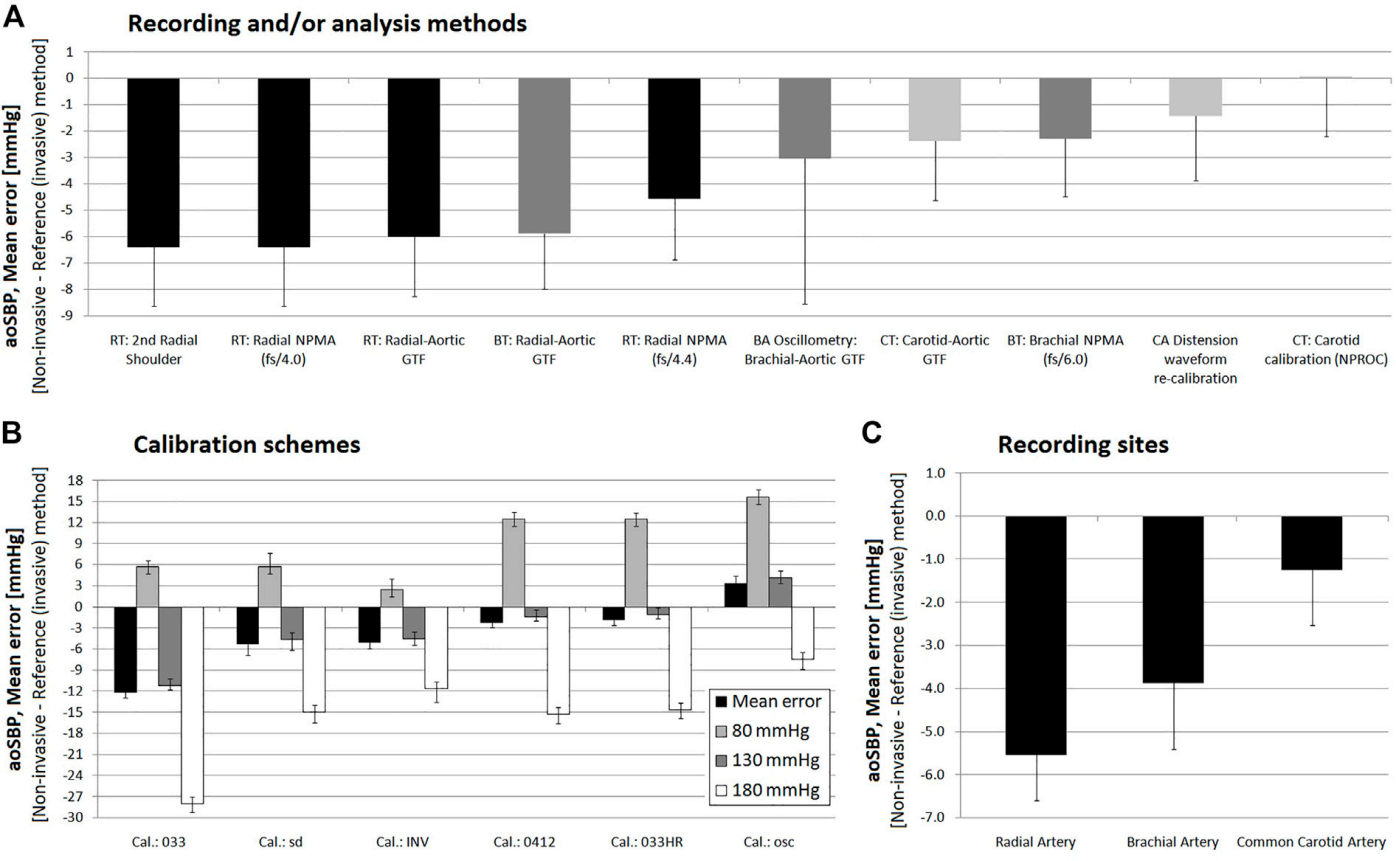
Pooled results for Bland-Altman derived mean errors (and lower or upper 95% confidence limits) obtained for comparisons between non-invasive and invasive aoSBP measurements. Results grouped according to: **(A)**, recording and/or analysis method; **(B)**: calibration scheme; **(C)**: recording site.

**FIGURE 8:**
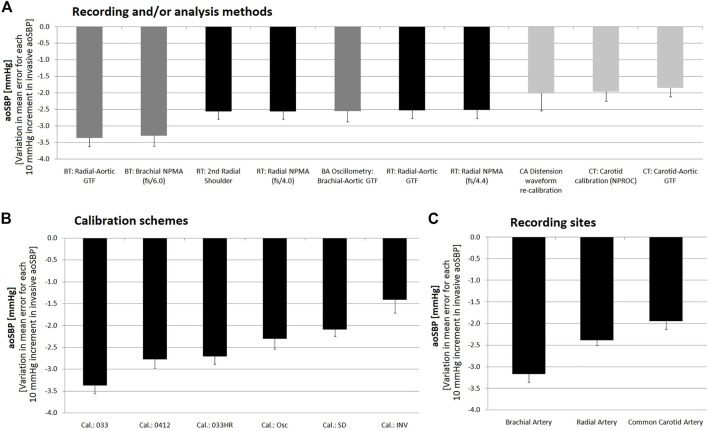
Pooled results for Bland-Altman derived proportional errors (and lower or upper 95% confidence limit) obtained for comparissons between non-invasive and invasive aoSBP measurements. Results grouped according to: **(A)**, recording and/or analysis method; **(B)**: calibration scheme; **(C)**: recording site.

**FIGURE 9:**
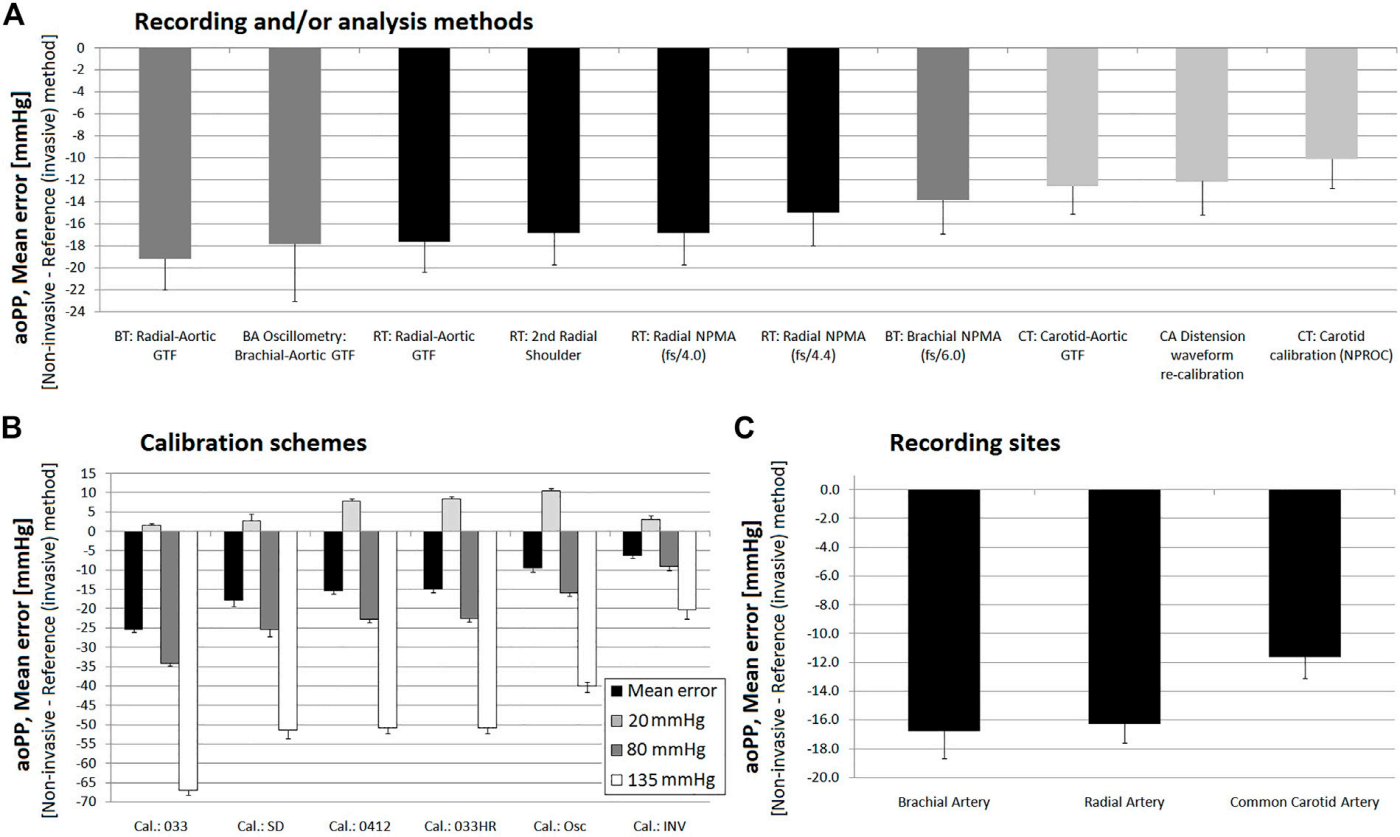
Pooled results for Bland-Altman derived mean errors (and lower or upper 95% confidence limit) obtained for comparisons between non-invasive and invasive aoPP measurements. Results grouped according to: **(A)** recording and/or analysis methodology; **(B)** calibration scheme; **(C)** recording site.

**FIGURE 10:**
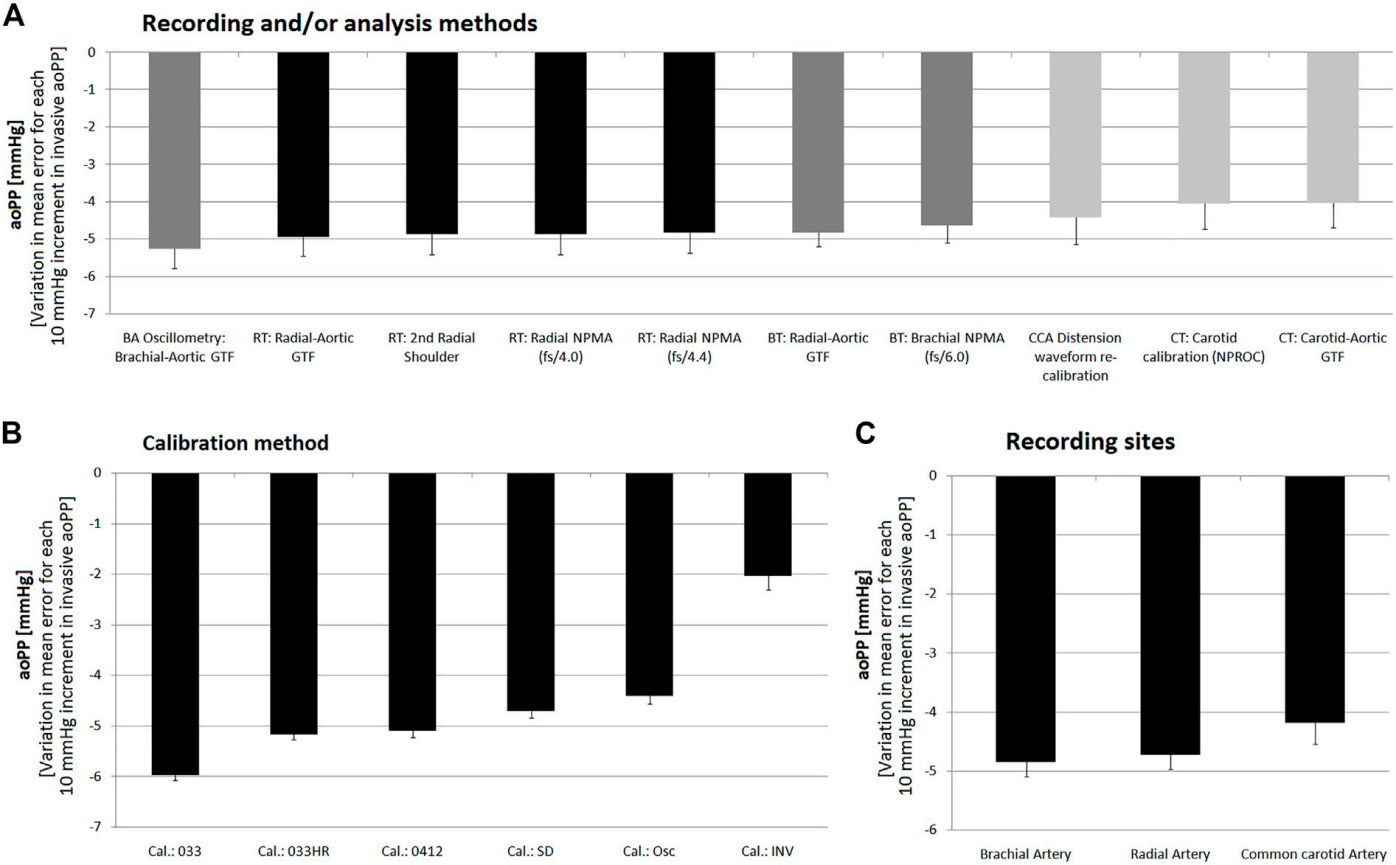
Pooled results for Bland-Altman derived proportional errors (and lower or upper 95% confidence limit) obtained for comparisons between non-invasive and invasive aoPP measurements. Results grouped according to: **(A)**, recording and/or analysis methodology; **(B)**: calibration scheme; **(C)**: recording site.

Figures 7, 8 (for aoSBP), and Figures 9, 10 (for aoPP) show the pooled results (errors derived from the Bland-Altman test), when considering: 1) the recording and analysis methodology (regardless of calibration scheme), 2) the calibration scheme (regardless of the recording and analysis methodology), and 3) the recording site (CCA, BA or RA).

Bland-Altman analysis correspond to the reference method (invasive aoSBP or aoPP data; *x*-axis) against the non-invasive and invasive difference (non-invasive minus invasive data; *y*-axis). The corresponding linear regression equations were obtained. Systematic error was considered present if mean error was significantly different from zero; proportional error was considered present if the slope of the linear regression was statistically significant. Considering the mean and proportional errors (regression equation) obtained from Bland-Altman analysis, the mean difference between: 1) invasive and 2) non-invasive derived aoSBP, and aoPP, was calculated (and graphed) for different aoSBP (Figures 4D, 7B) and aoPP (Figures 6D, 9B) values.

According to the central limit theorem, taking into account Kurtosis and Skewness coefficients distribution and the number of subjects (sample size >25) a normal distribution was considered (Lumley et al., 2002). Data analyses were done using MedCalc (v.14.8.1, MedCalc Inc, Ostend, Belgium) and IBM-SPSS Statistical Software (v.26, SPSS Inc, Illinois, United States). A *p* <0.05 was considered statistically significant.

## 3 Results

### 3.1 Population and hemodynamic characteristics

The group was characterized by a wide age range (61 ± 19 y, range: 14–89 y) with good sex distribution (Table 1). Descriptive information on hemodynamic characteristics can be seen in Table 2 and Supplementary File S2 (Tables S1–S5). Invasive bSBP values were distributed in a wide range: 6.5% of the analyzed population had <100 mmHg, 58.1% between 100 and 139 mmHg, 19.4% between 140 and 159 mmHg and 16.1% exhibited values ≥160 mmHg. On the other hand, the distribution of invasive bDBP values was: 19.3% < 60 mmHg; 71.0% between 60 and 84 mmHg and 9.7% > 85 mmHg. HR values were always within normal range.

### 3.2 Association between invasive and non-invasive aoSBP and aoPP

CCC levels obtained when analyzing the association between invasive and non-invasive aoSBP were between 0.7 and 0.9, indicating, strong, and very strong degrees of concordance or agreement. Overall, the lowest (but still statistically significant) CCC levels were obtained between invasive aoSBP data and the obtained non-invasively when calibrating to bMBP_033_ and bDBP (Figure 2A).

For aoPP, the highest CCC levels between invasive and non-invasive data were obtained when calibrating to bMBPosc. In turn, the lowest levels of association were obtained when calibrating to bMBP_0.33_ and bDBP (Figure 2B).

The strength of association between invasive and non-invasive recordings was higher when considering aoSBP than when considering aoPP (Figure 2).

### 3.3 Agreement between invasive and non-invasive aoSBP

Figures 3, 4 show mean and proportional errors (bias) and the 95% confidence interval (CI) obtained when comparing invasive and non-invasive aoSBP data. In each figure, values were ordered 1) from least (negative) to greatest (positive) value (A, left), 2) according to the calibration method (B, middle) and 3) considering the methodological approach (C, right). Additionally, Figure 7 shows mean errors’ pooled results when considering: 1) the recording and/analysis methodology, 2) the calibration scheme, and 3) the arterial recording site.

The different devices/techniques yielded a wide range of mean errors (−15.2 to 7.9 mmHg). Most of the approaches (42 out of 57) showed negative mean errors. In 32 (32/57) mean error values were between −5 and 5 mmHg and in 30 (30/32) of them the error was not statistically significant (Figure 3A).

The pooled analysis of the records showed that overall, irrespective of the 1) recording method (Figure 7A), 2) calibration scheme (Figure 7B), and 3) recording site (Figure 7C), non-invasive measurements underestimated aoSBP (with the sole exception of data obtained calibrating to bMBPosc/bDBP.

#### 3.3.1 Invasive vs. non-invasive aoSBP: Mean and proportional errors and recording and/or analysis method

Regardless of the calibration scheme, pooled results showed that aoSBP obtained from CCA waveform analysis (ultrasound or tonometry), followed by methods based on BA waveforms recordings were the ones that minimized mean errors (outperforming aoSBP values obtained from RA waves analysis) (Figure 7A). Sub-analysis of the methods showed that the different analyses that could be done from RA waves, did not show significant differences in terms of mean error with respect to invasively measured aoSBP (Figure 7A).

The “slopes” (proportional errors) of the linear adjustments made in the Bland-Altman analyses, showed that regardless of the calibration method, aoSBP data obtained from CCA records showed little change in mean error related to inter-individual differences in invasive aoSBP. In this regard, a 10 mmHg variation in invasive aoSBP was associated with a change in the error (difference between “non-invasive and invasive” data) equal to −2.01 ± 0.53 mmHg (method: CCA diameter waveform re-calibration), −1.96 ± 0.28 (method: CT, carotid calibration (NPROC)), and −1.84 ± 0.27 mmHg (method: CT, carotid-aortic GTF) (Figure 8A).

#### 3.3.2 Invasive vs. non-invasive aoSBP: Mean and proportional error and calibration scheme

Regardless of the recording and/or analysis method, the results showed that with the sole exception of the calibration based on bMBPosc, the calibration schemes resulted in lower non-invasive than invasive aoSBP levels (negative mean errors) (Figure 7B). The ‘033HR’ and “0412” calibration schemes (followed by the “sd” method) ensured the lowest mean errors. The calibration method using a bMBP_033_ generated the aoSBP levels furthest away from those recorded invasively (Figure 7B).

When evaluating the impact of the calibration method on the accuracy of non-invasive aoSBP levels, it was observed that calibrating to bMBPosc, bMBP_033HR_ or bMBP_0.412_ enabled obtaining mean error values closest to 0 mmHg (Figure 3A; Supplementary Table S8). On average, and regardless of the technique/method used, calibrating to bMBPosc, bMBP_033HR_ or bMBP_0.412_, resulted in mean errors equal to 3.4 mmHg, −1.9 mmHg and −2.3 mmHg, respectively (Figure 3A). Mean errors were between −5 and 5 mmHg when bMBP_0.33HR_ and bMBP_0.412_ calibrations were considered. When calibrating to bMBPosc, 3 out of 10 approaches showed mean bias ˃5 mmHg (Figure 3A). Then, calibrating to bMBPosc, bMBP_033HR_ or bMBP_0.412_ could be equally useful to minimize mean errors when assessing aoSBP.

For all the methodological approaches used, calibrating to bMBP_033_ (without considering HR) resulted in significant underestimation of aoSBP (mean error: −15.2 mmHg to −8.5 mmHg; “average” mean error: −12.2 mmHg). When calibrating to “sd,” the findings differed, depending on the device, technique and/or artery considered (Figure 3A; Supplementary Table S10). When obtaining aoSBP from CCA and BA recordings, calibrating to “sd” showed lower mean error levels, although not necessarily “better” than the obtained when calibrating to bMBP_033HR_ or bMBP_0.412_.

Calibrating to bMBP/bDBP did not necessarily minimize mean error. Then, factors not necessarily related to differences between invasive and oscillometric bBP would determine the agreement between invasive and non-invasive aoSBP (Figure 3A).

When proportional errors were analyzed, results showed that the higher the invasive aoSBP, the greater the underestimation achieved when using non-invasive approaches (Figures 3B, 7B). In this regard, calibrating to bBP, for each 10 mmHg increase in invasive aoSBP, the systems associated an underestimation that varied between −1.5 and −4.4 mmHg (Figure 3B).

Within the aoBP range considered in our study, most of the non-invasive approaches used overestimated and underestimated, respectively at at low (i.e., 80 mmHg) and high (i.e., 180 mmHg) aoBP values (Figures 3B, 7B). Calibration using bMBP_033_ (without HR adjustment) resulted in the highest underestimation levels at high aoSBP values (−28.06 mmHg) while the calibration to bMBPosc showed the lowest underestimation (Figures 3B, 7B). For calibrations to bMBP_033HR_ or bMBP_0412_ the errors were distributed more homogeneously around 0 mmHg (Figures 3B, 7B).

Analysis of proportional errors (“slopes”) showed that calibration to bMBP_033_ was the scheme that resulted in the largest differences (3.37 ± 0.18 mmHg) between invasive and non-invasive data (for 10 mmHg variations in invasive aoSBP values). The other calibration methods showed smaller variations, with the calibration to invasive bBP values showing the smallest slope (Figure 8B).

#### 3.3.3 Invasive vs. non-invasive aoSBP: Mean and proportional error and recording site

In agreement with what has been already mentioned, non-invasive aoSBP assessment based on CCA (followed by BA) recordings allowed minimizing mean error respect to invasive aoSBP (regardless of the recording/analysis method and calibration scheme) (Figures 3C, 7C).

Additionally, the methods considering CCA recordings showed the least error sensitivity (between non-invasive and invasive records) against variations in invasive aoSBP (Figure 8C).

### 3.4 Agreement between invasive and non-invasive aoPP

Figures 5, 6 show, respectively, mean and proportional errors, together with the 95% CI obtained when comparing invasive and non-invasive data for aoPP. Additionally, Figures 9, 10 show, respectively, mean and proportional errors’ pooled results when considering the: 1) recording and/analysis methodology, 2) calibration scheme, and 3) recording site.

The different approaches yielded a wide range of mean errors (−30 to −3 mmHg) when the agreement between non-invasive and invasive aoPP was analyzed. All approaches (*n* = 57) showed negative mean errors and only three did not reach statistical significance, despite showing a mean errors of −4.86, −3.45 and −3.58 mmHg) (Figure 5). On the other hand, only aoPP_SCOR_CT_NPRO_inv showed mean errors whose CI were within +5 and −5 mmHg (Figure 3A).

#### 3.4.1 Invasive vs. non-invasive aoPP: Mean and proportional errors and recording and/or analysis method

Regardless of the calibration scheme, the aoPP levels obtained from CCA waves analysis were those that minimized mean error (Figure 9A), although in all cases it was ≥10 mmHg.

When analyzing the “slopes” (proportional errors), regardless of the calibration method, aoPP levels obtained from CCA records were the which showed less change in mean error related to inter-individual variations in invasive aoPP. In fact, for CCA-derived non-invasive methdos, a variation of 10 mmHg in the invasive aoPP was associated with a change in error (difference between “non-invasive and invasive” record) of aproximately −4.0 mmHg (Figure 10A).

#### 3.4.2 Invasive vs. non-invasive aoPP: Mean and proportional error and calibration scheme

Irrespective of the recording and/or analysis method, pooled results showed that all calibration schemes resulted in aoPP levels lower than those recorded invasively (negative mean error) (Figure 9B). However, similar to that reported for aoSBP, calibration methods using oscillometry derived bMBP and those that quantified bMBP using a form factor equal to “0.412” and/or “0.33” corrected for heart rate (‘033HR’) achieved smaller errors than calibration using a form factor of “0.33” without HR correction (Figure 9B). Furthermore, the latter resulted in mean error close to 25 mmHg.

The higher the invasive aoPP, the greater the underestimation achieved when using non-invasive approaches (Figure 6D). When low invasive aoPP levels were considered, depending on the non-invasive approach analyzed the data obtained underestimated, overestimated or were similar to the aoPP invasively measured (Figure 6D).

The analysis of the “slopes” (proportional errors), showed that the calibration to bMBP_033_ was the one resulted in the greatest differences (−6.0 ± 0.1 mmHg) between invasive and non-invasive aoPP, for 10 mmHg variations in invasive aoPP. Calibration with invasive bBP provided an error that remained almost unchanged as aoPP levels varied (Figure 10B).

#### 3.4.3 Invasive vs. non-invasive aoSBP: Mean and proportional error and recording site

Regardless of the recording method, analysis methodology and/or calibration scheme considered, aoPP measurements based on CCA-derived data were the ones that allowed minimizing the mean error with respect to invasive aoPP (Figures 5C, 9C). In addition, these methods had the lowest error sensitivity to variations in invasive aoPP levels (Figure 9C).

## 4 Discussion

There are still several questions regarding the accuracy and validity of approaches and devices used for non-invasive aoBP estimation that await answers. Their optimal mode of application could, in theory, depend on factors such as the methodology, recording site and the calibration scheme considered. In this context, in this study, using validated devices, we analyzed how these factors may interact when determining aoSBP and aoPP. We specifically evaluated accuracy of the devices considering different calibration schemes (bSBP/DBP or bMBP/DBP), pulse waveform acquisition techniques (applanation tonometry, oscillometry, vascular ultrasound) and arterial recording sites (RA, BA, and CCA). The main results are discussed step by step, highlighting complementary issues.

### 4.1 Association between invasive and non-invasive aoBP

• First. Disregard of the recording and analysis methodology, calibration scheme and/or recording site, non-invasively obtained aoSBP and aoPP levels were associated with those obtained invasively. However the strength of the association 1) was higher when considering aoSBP and 2) reached the lowest levels when using the bMBP_033_/bDBP calibration scheme.

The above is in agreement with other authors’ findings. About this, in patients (*n* = 50; age: 62.4 ± 8.9 years) without cardiac disease, Chi et al. (2022) measured invasive and non-invasive aoSBP using three devices (SphygmoCor, Mobil-O-Graph, PulsePen) and different schemes of waveforms calibration (bSBP/bDBP and bMBP/bDBP—unfortunately the authors did not explain how the bMBP was obtained). Like in this work, the authors found significant positive associations (regardless of the device and calibration method) between invasive and non-invasive aoSBP, and levels of association strengths (r) between 0.63 and 0.72 (somewhat lower than those found in this study). In turn, Negishi et al. (2016) reported modest levels of association (r = 074) between aoSBP obtained using the Mobil-O-Graph calibrated with bSBP/bDBP and bMBPosc/bDBP. Then, the correlation values found in our work agree with those previously reported.

### 4.2 Agreement between invasive and non-invasive aoSBP: Mean and proportional error

• Second. Overall, disregard of the methodology, calibration scheme and/or recording site, non-invasive approaches resulted in aoSBP levels lower than those recorded invasively. However, 56% of the approaches (32 out of 57), yielded mean errors close to 0 mmHg (distributed between −5 and +5 mmHg).

The above described is in agreement with previous works that described that in general terms, non-invasive measurement approaches underestimate invasive aoSBP levels. In a meta-analysis that included studies in which aoBP was measured invasively and non-invasively using applanation tonometry, Cheng et al. (2013) found that when calibrating peripheral waves with invasive bBP, mean errors for aoSBP (16 studies, 764 measurements), and aoPP (8 studies, 395 measurements) were: −1.1 ± 4.1 mmHg (95% CI: −9.1 to 6.9 mmHg) and −0.8 ± 5.1 mmHg (95% CI: −10.8 to 9.2 mmHg), respectively. However, when the signals were calibrated to bBP, the mean errors scaled to −8.2 ± 10.3 mmHg (95% CI: −28.4 to 12.0 mmHg) and to −12.2 ± 10.4 mmHg (95% CI: −32.5 to 8.1 mmHg) when considering aoSBP and aoPP, respectively. In addition, when data from devices “clearly” validated for bBP measurement (“validated cuff bBP monitors”) were considered, the results (mean errors) remained almost unchanged: −6.7 ± 10.6 mmHg (95% CI: −27.4 to 14.1 mmHg) for aoSBP and -15.0 ± 11.1 mmHg (95% CI: −36.7 to 6.6 mmHg) for aoPP.

In a systematic review and meta-analysis (22 eligible studies, 11 commercial devices and 808 subjects), aimed at determining the accuracy of commercially-available validated devices used to estimate aoSBP non-invasively, Papaioannou et al. (2016) reported that, in general, non-invasive devices underestimated invasive aoSBP (mean error: −4.49 mmHg; 95% CI: −6.06 to −2.92 mmHg) (Papaioannou et al., 2016-A). On the other hand, the pooled error for the studies that calibrated the devices using invasively recorded bBP values (*n* = 11) was not statistically different from 0 (mean error = −1.08 mmHg, 95% CI: −2.81 to 0.65 mmHg), although the individual analysis showed that there were significant errors in 6 (4 underestimated and 2 overestimated). In contrast, when calibration was done using non-invasively measured bBP values, the error estimate (for aoSBP) was −5.81 mmHg (95% CI: −7.79 to -3.84 mmHg); significantly different from 0 mmHg (*p* <0.001).

In agreement with the above, in this work we found that non-invasive methods used to determine aoSBP generally underestimated its value.

• Third, pooled results showed that determinations based on CCA recordings (by ultrasound or tonometry), followed by BA recordings, were those that yielded aoSBP values closest to those recorded invasively (Figures 7A, C). In addition, regardless of the calibration method, aoSBP levels obtained from CCA data showed the least change in error related to inter-individual variations in invasive aoSBP (Figures 8A, C). On the other hand, there were no differences in mean error levels obtained with different methodological approaches based on RA recordings (i.e., radial-aortic GTF, RA second shoulder, NMPA).

Taking into account that described above, it could be said that the recording site could be one of the main determinants of the ability of a non-invasive method to assess invasive aoSBP values. Our results allow proposing that there is a hierarchical order in terms of ability to accurately quantify real aoSBP values: CCA ˃ BA ˃ RA. The “hierarchical” order found in this work (in which multiple measurements were obtained in a single individual) is in agreement with what arises from the work carried out by Papaioannou et al. (2016). The authors reported that when peripheral waves were acquired from RA and then calibrated to bBP values measured either invasive or non-invasively, the pooled error in aoSBP estimation was −5.41 mmHg (95% CI: −7.59 to −3.23 mmHg). In turn, when the waveform was recorded at the BA the estimated error was −3.22 mmHg (95% CI: −5.53 to −0.91 mmHg). In contrast, when CCA waves were recorded, the estimated error was not significantly different from 0 mmHg (mean error: −2.35 mmHg; 95% CI: −10.49 to 5.78 mmHg). So, it could be said that the error gradually increased from CCA (−2.35 mmHg), followed by BA (−3.22 mmHg) and finally reaching RA (−5.41 mmHg).

• Fourth, when considering the calibration scheme, pooled results showed that disregard of the recording and/or analysis method and recording site, the different calibrations resulted in aoSBP levels lower than those recorded invasively (negative mean error), with the sole exception of the calibration to bMBPosc, (Figure 7B). The calibration schemes considering “033HR” and “0412” ensured the lowest mean error, whereas the calibration considering “033” resulted in aoSBP levels furthest away from those recorded invasively (Figure 7B).

One of the main problems regarding the calibration procedure is bMBP estimation. The only way to measure bMBP is by intra-arterial catheterization, but several methodologies have been proposed to estimate MBP non-invasively, in order to calibrate RA, BA or CCA waveforms and determine aoSBP and aoPP (Agnoletti et al., 2012). Discussion of this issue requires at least three separate analyses.

First our results showed that the way bMBP is quantified is an important determinant of the aoSBP levels non-invasively obtained. Then, the level of agreement between non-invasive and invasive aoSBP data would vary (significantly) depending on the approach chosen to quantify bMBP. Several works stated that bMBP/bDBP calibration outperforms bSBP/bDBP (“sd”) calibration in the quantification of aoSBP. However, looking at our results this would not be always the case, but it would depend on the method used to determine bMBP. About this, it is to note that, unfortunately, the way to obtain bMBP is not specified. In the systematic review and meta-analysis conducted by Papaioannou et al. (2016), it was reported that when bSBP and bDBP were used for calibration (“sd calibration”), the estimated error was −7.78 mmHg (95% CI: −10.28 to −5.28 mmHg), whereas the joint estimate of the error in the aoSBP estimate was −2.99 mmHg (95% CI: −5.76 to −0.22 mmHg) when calibrating to bMBP/bDBP. Unfortunately, the authors did not analyze the way bMBP was obtained. Similarly, it has been reported that different calibration methods (especially bSBP/bDBP and bMBP/bDBP) did not result in differences in aoSBP values that could be estimated, but they did not describe how bMBP was quantified (which would enable a comparative analysis with our results) (Chi et al., 2022).

Consequently, calibrating using bMBP/bDBP would outperform calibrating using bSBP/bDBP, only when using some methods to quantify bMBP.

Calibration to systolic-diastolic (“sd”) levels showed dissimilar results depending on the device, technique and/or artery considered which could contribute to explain the contradictory findings reported in the literature. For example, when calibrating using the “sd” scheme for data obtained from BA oscillometry (Mobil-O-Graph) and RA tonometry (SphygmoCor), mean errors were within −10.1 and 8.5 mmHg (statistically significant). In turn, for data obtained from BA or CCA using tonometry and/or ultrasonography the bias were between −4.3 and 1.7 mmHg (without statistical significance) (Figure 3A; Supplementary Table S10).

As a second issue that arises from our results is that calibration schemes using “033HR” and “0412” led to lower errors than those achieved when using a 33% form factor (without HR correction). This shows that small errors in bMBP quantification could have a large impact on aoSBP levels obtained non-invasively. The approach most commonly used in clinical practice is to derive bMBP using the form factor equal to 33% (bMBP = bDBP+1/3*bPP). However, the applicability and accuracy of this simple formula has been questioned. Bos et al. reported that 40% of bPP (instead of the 33% used in the one-third rule) should be added to bDBP (Bos et al., 2007). Indeed, it was proposed that the difference (FF equal to 33% versus 40%) could have an important impact on the non-invasive assessment of aoBP (Mahieu et al., 2010; Papaioannou et al., 2016). In Mahieu et al. (2010) work, which compared aoSBP levels obtained with three radial and carotid wave calibration schemes, as well as in the systematic review and meta-analysis done by Papaioannou et al. (2016), it was reported that compared to the use of a FF equal to 33%, the use of a FF of 40% or 41.2% in the calibration scheme used to obtain bMBP resulted in aoSBP levels closer to (but lower than) those obtained invasively. In addition, the calculation of bMBP using 0.412 was superior to the traditional formula using 33% in discriminating subjects with cardiac and carotid wall hypertrophy, as well as subjects with increased aortic stiffness (Papaioannou et al., 2016-B).

As a third and important issue to consider, it is to note that calibration to invasive bBP values did not always result in the lowest differences between non-invasive and invasive aoSBP data. This could be because the mathematical analysis procedures included in each algorithm were developed tailored for routine laboratory and/or hospital work, which mostly involves signal calibration based on non-invasive bBP recordings (e.g., cuff devices), recognized as different from invasive ones. Related with this, previous reports have postulated that, in general, non-invasive methods used to determine bBP, overestimate diastolic and underestimate systolic and pulse bBP invasively obtained (Papaioannou et al., 2016-A; Picone et al., 2017; Kobayashi et al., 2013; Jedrzejewski et al., 2021; Cheng et al., 2013; Shih et al., 2011). The reason for this is that international standards for bBP monitors require manufacturers to validate bBP monitors tested with the mercury cuff method using Korotkoff sounds, which is actually not an accurate method for measuring bBP when compared to intra-arterial bBP (Smulyan and Safar, 2011; Cheng et al., 2013).

• Fifth, when errors (difference between non-invasive and invasive data) were analyzed in relation to invasive aoSBP levels, most non-invasive approaches overestimated and underestimated aoSBP at low (i.e., 80 mmHg) and high (i.e., 180 mmHg) pressure levels, respectively (Figures 3B, 7B, 8). Calibration to bMBP_033_ (without HR adjustment) resulted in the highest levels of underestimation (-28.06 mmHg), whereas calibration to bMBPosc and either bMBP_033HR_ or bMBP_0412_ led to smaller and more homogeneously distributed (around 0 mmHg) errors (Figures 3B, 7B).

The finding of proportional errors when analyzing the agreement between non-invasive and invasive aoSBP recordings, opposes to that reported by Chi et al.(2022) who analyzed data obtained with SphygmoCor, Mobil-O-Graph and PulsePen. On the contrary, results obtained by Cai et al. (2021), in children and adolescents (*n* = 29, age: 2–16 years), conceptually agree with our findings. In this regard, the authors reported that as invasive aoSBP levels were gradually reduced, non-invasive methods tend to increasingly overestimate aoSBP levels (RA and CCA tonometry, SphygmoCor; BA oscillometry/plethysmography, SphygmoCor) (Cai et al., 2021), which is in line with our results. It is to note that all the children included in Cai et al. work had aoSBP levels (range 70–100 mmHg) that would correspond to the lowest values recorded in the adults included in this work. Thus, results from both Cai et al. work and this one support the existence of proportional bias, such that low invasive aoSBP levels (in children, adolescents or adults) would be overestimated by non-invasive approaches (while the opposite would be observed at high aoSBP levels).

• Sixth, regardless of the methodology and/or the calibration scheme considered, non-invasive aoSBP data obtained from CCA recordings were the closest to invasive aoSBP values (Figures 3C, 7C). In addition, the methods that support their approach on CCA recordings showed the least error sensitivity to variations in invasive aoSBP (Figure 8C).

As mentioned above, whichever approach is chosen, an important issue is to record “as close to the aorta as possible”, taking into account a hierarchical order (CCA ˃ BA ˃ RA) would be in terms of accuracy in central pressure estimation. The possible superiority of methods based on CCA recordings over those based on RA or BA recordings is in agreement with recent (2022) findings of Chi et al. (2022) The authors reported that, compared to approaches based on data from RA (tonometry, SphygmoCor) and BA (oscillometry, Mobil-O-Graph), the method based on CCA applanation tonometry was the only one that did not tend to underestimate aoSBP.

### 4.3 Agreement between invasive and non-invasive aoPP: Mean and proportional errors

As a seventh issue, it is to note that findings related with aoPP recordings were mostly in line with that already mentioned for aoSBP. All approaches (*n* = 57) showed negative mean errors when comparing non-invasive and invasive data (invasive aoPP underestimate). In addition, a wide range of mean errors was observed (−30 to −3 mmHg) (Figure 5A). The higher the invasively measured aoPP the higher the underestimate provided by non-invasive methods. Regardless of the recording and/or analysis method, pooled results showed that all calibration schemes resulted in lower aoPP levels than those recorded invasively (negative mean error) (Figure 9B). However, like it was observed for aoSBP, calibration methods using bMBPosc and those quantifying bMBP using a form factor equal to “0.412” and/or “0.33” corrected for HR (“033HR”) resulted in the least bias (Figure 9B). On the other hand, using the “033” calibration scheme, the mean error achieved 25 mmHg, at the time the highest proportional error levels were achieved (error: −6.0 ± 0.1 mmHg).

Finally, as reported for aoSBP, regardless of the calibration scheme considered, aoPP levels obtained from CCA waveforms analysis minimized mean errors (although they were not negligible) and resulted in the lowest proportional bias.

### 4.4 Physiological and clinical relevance

The potential explanatory factors and meaning of this work’s findings should be analyzed and highlighted. First, we found that overall, disregard of the technology/methodology, calibration scheme and recording site considered non-invasive approaches resulted in aoBP levels lower than those recorded invasively. This would have several connotations when analyzing and understanding the central hemodynamics. In this regard, as an example, it should be noted that aoBP levels considered to quantify left ventricle wall stress during ejection (afterload) would conduct to an underestimate of the actual values (Zócalo et al., 2013). Then, the understanding of physiological and/or pathological phenomena such as the relationship between cardiac wall stress and wall/lumen remodeling or systolic/diastolic function would be innacurately evaluated. Second, it shows that the values of aoSBP and aoPP that we use for different calculations related to arterial function and/or haemodynamic states (e.g., compliance, distensibility or pressure-strain elastic modulus to evaluate local arterial stiffness) would be associated with (or result in) errors when calculating biological parameters. About this, it is to note that for a given change in arterial pulsatile diameter, the corresponding aoPP would be higher than the estimated, and consequently the central arteries may be actually stiffer than what we usually consider. In turn, the above would “impact” on the recognized differences in arterial stiffness (and pulse pressure wave) when considering central and peripheral arteries. Third, the fact that aoBP values would not be obtained accurately (and additionally that data would differ depending on the approach considered) could influence (distort) the relationship between aoBP and cardiovascular risk levels, at the time it could contribute to explain differences in available data regarding the clinical value of aoBP in terms of risk stratification. Finally, 1) the differences (systematic and/or proportional errors) between methods, recording sites and/or calibration schemes in terms of aoSBP and/or aoPP values obtained, and 2) the existence of levels of over–and underestimation at low and high invasive aoBP levels, respectively, show the need for technological and/or methods improvements, since it is currently difficult to systematize (or simplify) the error that could be found when performing measurements in a given subject.

Second, we found that the closest approximation between invasively and non-invasively measured aoBP was obtained when considering CCA recording (regardless of the method used). This could be related (explained) to the fact that carotid records do not require the use/application of specific wave propagation models (e.g., GTFs), generally derived from population studies and which could not adjust to the specificity of the patient evaluated, but simply assume similarity in BP levels and waveforms between the aorta and carotid arteries (due to their anatomical proximity). On the other hand, our finding allows to postulate that, if possible, direct records from CCAs should be attempted (prioritized) when aiming at quantifying aoBP values non-invasively. However, high-quality CCA records are not always possible to obtain (e.g., in very thick necks, in subjects with respiratory disorders, in neonates or infants) and alternative recordings are necessary. In this cases, peripheral waveforms records and different methodological approaches (e.g., application of GTFs) can be used to obtain (estimate) aoBP. It is to note that the estimation of aoBP from peripheral recordings is what allowed the development of portable devices that quantify aoBP on an ambulatory basis (e.g., Mobil-O-Graph). Anyway, our results show that the degree of agreement between data from these devices and the obtained invasively would be lower than the achieved when evaluating carotid arteries. The above should be considered when using such valuable devices.

Third, we found that when calibrating the BP waveforms, using non-invasively measured bSBP and bDBP levels, a form factor of approximately 0.40 minimized the error between aoBP levels obtained non-invasively and invasively. Additionally, our results show significant calibration-dependent differences in the aoBP levels (even for the same device!). This demonstrates the importance of 1) being able to agree as soon as possible (consensus) on the form factor that should be used to calibrate the aoBP records, and 2) (authors) communicating the scheme of calibration used. This is necessary (essential) to be able to evaluate, analyze and compare adequately data obtained in different studies and/or populations.

### 4.5 Strengths and limitations

Our results should be analyzed in the context of the work’s strengths and limitations.

First, like most of this kind of studies (e.g., those included in the aforementioned meta-analysis (Cheng et al., 2013; Papaioannou et al., 2016-A)), the present work was not conducted in healthy subjects, as an invasive study is only indicated for clinical reasons in the context of suspected or known cardiovascular disease. Nevertheless, the subjects included in this study would be considered representative of those in whom an accurate knowledge of cardiovascular variables would be of critical clinical importance (e.g., aoSBP in the context of assessing target organ damage associated to hypertension, to quantify risk, and/or to evaluate therapy).

Second, we studied 34 subjects. Although the sample size can be considered moderate, it should be noted that it was sufficient to detect statistical differences and, consequently, achieved satisfactory statistical power (avoiding a type 2 statistical error). The invasive recordings in the contra-lateral BA, as well as the second invasive recording at the level of the aortic root, were part of the research protocol and not of the medical diagnostic evaluation (catheterization for diagnostic purposes). The same consideration applies to the non-invasive oscillometric, echographic and tonometric recordings. These recordings increased the duration of catheterization by at least 30–40 min. Consequently, this greatly limited the number of people who that could be elected and/or agreed to be included in this study. Then, 34 subjects is an important sample size for a study that aims to demonstrate the relevance of several issues, but not necessarily to be conclusive on this important topic that will necessarily require further study. Anyway, it is recognized that the number of patients studied is not large and results may not be extensible or extrapolated to the general population. In this context, it should be noted that most of the studies like this one considered sample sizes smaller, equal or slightly larger than the included in this work (Cheng et al., 2013; Papaioannou et al., 2016-A).

The differences between invasive and non-invasive BP levels could vary in association with factors like age, anthropometric characteristics, sex, smooth muscle reactivity (Bia et al., 2008; Curcio et al., 2016). However, our sample was not enough large and/or heterogeneous to allow defining subgroups (e.g., considering subjects age, sex and/or exposure to risk factors) and to be able to apply statistical tests with sufficient power. Future, multicentre studies, allowing for the inclusion of a large number of subjects will be necessary to assess the impact of covariates or confounding factors on the results (e.g., sex- and/or age-stratification analyses).

Third, we used “fluid column” pressure transducers instead of solid-state pressure sensors. Clearly, solid-state sensors are characterized by a higher accuracy in obtaining the BP waveform, mainly because they are able to detect high-frequency components. However, fluid column transducers are widely used in clinical practice to obtain aoSBP levels, and are used in our University Hospital. In this regard, it should be noted that the ARTERY Society task force consensus statement on protocol standardization (“Validation of non-invasive central blood pressure devices”), Sharman et al. (2017) state that although micromanometer-tipped catheters are the preferred instruments to use, meticulously managed fluid column catheters may also be acceptable to accurately measure intra-arterial BP. On the other hand, in the systematic review and meta-analysis done by Papaioannou et al. (2016), it was reported that mean errors in non-invasive estimation of aoSBP were similar when comparing fluid-filled and catheter-tipped transducers. Of course, the low-cost liquid-filled catheter manometer systems require much more cautious handling and operation (e.g., in terms of calibration, frequency response, positioning, zeroing) compared to the costly high-fidelity micro-tipped catheters. However, it should also be recognized that the use of liquid-filled manometer-catheter systems (if proven accurate), should be limited only to the assessment of maximum and minimum values of the pressure wave due to the damping of the wave characteristics. Thus, high-fidelity micromanometer-tipped probes should be used in studies aimed at assessing the validity of waveform-derived indexes (e.g., augmented pressure or index). In this context, considering the levels of natural frequency and damping coefficient of our catheter-tubing-external transducer system, and despite using widely validated equipment and measurement methodologies, it is clear that the invasively obtained peak systolic and minimum diastolic pressure levels may have been slightly over- and under-estimated, respectively.

Fourth, in our work, all comparisons between invasive and non-invasive recordings were made considering data obtained simultaneously or in immediate sequential form. This allowed to be sure about the correspondence between non-invasive and invasive recordings, avoiding possible time differences that could affect the results, as in the case of other works (e.g., comparing invasive and non-invasive recordings obtained up to 10 min apart) (Chi et al., 2022).

Fifth, non-invasive techniques based on echography and applanation tonometry are operator dependent. A non-experienced operator may inevitably render the estimates less reliable, less accurate and/or even invalid. In this work measures were performed by trained professionals (Y.Z, D.B.) (Zócalo and Bia, 2021 and, Zócalo and Bia, 2022 and Zócalo, 2021).

## 5 Conclusion

With regard to aoSBP. First, overall, non-invasive approaches yielded lower aoSBP levels than those recorded invasively. However, 56% of the approaches yielded global mean errors close to 0 mmHg (distributed between −5 and +5 mmHg). Most approaches overestimated and underestimated aoSBP at low and high invasive aoSBP levels, respectively. Second, irrespective of the recording and/or analysis method and recording site, “033HR” and “0412” calibration schemes led to the lowest mean errors, whereas the “033” method resulted in aoSBP levels furthest from those recorded invasively. Third, determinations based on CCA recordings (ultrasound or tonometry), followed by BA recordings, resulted in aoSBP values closest to those recorded invasively. Regardless of the calibration method, aoSBP levels obtained from CCA recordings showed the least changes in error related to inter-individual variations in invasive aoSBP.

With regard to the aoPP. First, all the approaches showed negative mean errors (underestimation of aoPP). The higher the invasive aoPP, the higher the underestimation given by non-invasive methods. Second, calibration methods using bMBPosc and bMBP_0.412_ and/or MBP_033HR_ led to the least mean errors. Third, regardless of the calibration scheme, aoPP levels obtained from CCA-derived records showed the least mean and proportional errors.

The results allow concluding that 1) the recording method and site; 2) the mathematical model used to quantify aoBP, and 3) the calibration scheme, are important at the time of estimating aoBP and should be considered when analyzing and/or comparing data from different works, populations and/or obtained with different approaches. The above highlights the need for methodological transparency and consensus for an accurate non-invasive aoBP assessment, which would contribute to increase its validity and value in both clinical and physiological research settings.

## Data Availability

The raw data supporting the conclusion of this article will be made available by the authors, without undue reservation.

## Glossary

**95% CI**: 95% confidence interval
**aoBP**: Central aortic blood pressure
**aoPP**: Central aortic pulse pressure
**aoSBP**: Central aortic systolic blood pressure
**BA**: Brachial artery
**bBP**: Brachial artery blood pressure
**bDBP**: Brachial artery diastolic blood pressure
**bDBP(Osc)**: Brachial artery diastolic blood pressure obtained with the oscillometric system
**bMBP**: Brachial artery mean blood pressure
**bMBPosc**: Brachial artery mean blood pressure obtained with the oscillometric system
**BP**: Blood pressure
**bPP**: Brachial artery pulse pressure
**bPP(Osc)**: Brachial artery pulse pressure obtained with the oscillometric system
**bSBP**: Brachial artery systolic blood pressure
**bSBP(Osc)**: Brachial artery systolic blood pressure obtained with the oscillometric system
**BT**: Brachial artery applanation tonometry
**CCA**: Common carotid artery
**CCC**: Lin’s Concordance Correlation Coefficient
CM: Calibration using calculated bMBP
**CRFs**: Cardiovascular risk factors
**CT**: Common carotid artery applanation tonometry
**Echo**: Echography or vascular ultrasound
**ExpAdj**: Use of an exponential fit for the diameter-pressure transformation
**FF**: Form factor
**GTF**: Generalized transfer functions
**HR**: Heart rate
**HR(Osc)**: Heart rate obtained with the oscillometric system (Mobil-O-Graph device)
**ICC**: Intraclass correlation coefficient
**MOG**: Mobil-O-Graph: oscillometry/plethysmography
**NPMA**: N-point moving average
**NPROC**: Not-processed approach´
**OScM**: Calibration using oscillometric-measured bMBP
**RA**: Radial artery
**RT**: Radial artery applanation tonometry
**SBP2**: Second peak or shoulder of the radial artery pulse waveform
**SCOR**: SphygmoCor applanation tonometry
**SD**: Systo-diastolic calibration
