# Supplementary material for: Aortic systolic and pulse pressure invasively and non-invasively obtained: Comparative analysis of recording techniques, arterial sites of measurement, waveform analysis algorithms and calibration methods
Source: Front Physiol. 2023 Jan 16;14:1113972. doi: 10.3389/fphys.2023.1113972 (PMC9885133; doi:10.3389/fphys.2023.1113972)
Supplement: Supplementary file 1 [file DataSheet1.PDF]

Supplementary Material

Aortic systolic and pulse pressure invasively and non-invasively obtained: comparative analysis of recording techniques, arterial sites of measurement, waveform analysis algorithms and calibration methods

Daniel Bia[1*#], Yanina Zócalo[1#], Ramiro Sánchez[2], Gustavo Lev[3], Oscar Mendiz[3], Franco Pessana[4], Agustín Ramirez[5], Edmundo I. Cabrera-Fischer[5]

*** Correspondence:** Daniel Bia, dbia@fmed.edu.uy; Yanina Zócalo, yana@fmed.edu.uy

1 Methods

1.1 Subjects

Thirty-four subjects (41% females; 14-89 years-old) with coordinated coronary angiogram at the Favaloro Foundation University Hospital were included (Sánchez et al., 2020). Subjects with valvular heart disease and/or arrhythmia were excluded. A brief clinical interview, together with an anthropometric evaluation, enabled to assess the exposure to cardiovascular risk factors (CRFs), defined according to criteria previously described (Zócalo and Bia, 2021-A, 2021-B; Zócalo and Bia, 2022; Zócalo et al., 2021; Bia and Zócalo, 2021). Body weight (Omron HBF-514C, Omron Healthcare, Inc., Illinois, USA) and height (portable stadiometer) were measured with the participants wearing light clothing and no shoes. Body mass index was calculated as body weight-to-squared height ratio. Laboratory biochemical data were obtained and echocardiographic examinations were performed (Table 1).

A specialized nurse gave patients a general explanatory guidance on the invasive procedure to be performed. Prior to examination, written informed consent was obtained from the participants and/or their parents. Informed assent was obtained when necessary. The study protocol was approved by the Institutional Ethic Committee and all procedures were performed in agreement with the Declaration of Helsinki.

The following evaluations (recordings) were performed in each subject: (1) invasive (vascular catheterization) measurement of aoBP and bBP levels and waveforms, (2) non-invasive assessment of aoBP levels and waveforms using: (i) applanation tonometry (SphygmoCor device) recordings of the CCA, BA and RA level, (ii) oscillometric/plethysmographic device (Mobil-O-Graph device) recordings from the BA and (iii) vascular ultrasound (SonoSite device) recordings of the CCA (Figure 1). Non-invasive recordings (SphygmoCor, Mobil-O-Graph and Sonosite Ultrasound device) were obtained in random order to avoid potential bias because of the time intervals between non-invasive and invasive measurements.

1.2 Invasive Measurement of Central Aortic and Brachial Artery Blood Pressure

Intra-arterial aoBP and bBP levels and waveforms were obtained with the subjects lying in the supine position, according to routine clinical practice and guidelines for invasive coronary arteries assessment. Briefly, standard asepsis was performed in the arterial access area (radial) followed by cutaneous/subcutaneous injection of lidocaine to minimize patient's pain and discomfort. A soft sedation (midazolam 1.5 mg and fentanyl 0.025 mg) was also administered as needed. After local anesthesia was applied in the vessel access area, a 5 or 6 French introducer sheath was positioned in the arterial lumen and heparin (5,000 units) was administered through the arterial catheter. Subsequently, a 0.035-inch guide wire was advanced and placed in the ascending aorta and, a 5 French pig tail catheter (Cordis, Miami, USA) was introduced thereafter. Special attention was paid to place the pig tail catheter tip ~4 cm away from the aortic valve. After confirming the correct positioning of the catheter, which was assessed visually via fluoroscopy (Allura Xper FD10 or AlluraClarity FD20/10, Philips Healthcare, the Netherlands), the guide wire was removed and the intra-arterial catheter was flushed with saline solution.

To record intravascular pressure, the aforementioned fluid-filled catheter placed in the proximal ascending aorta (or brachial artery) was connected to the external blood pressure transducer (MX960, Medex, LogiCal, Smiths Medical ASD Inc., Minneapolis, USA), and the transducer was connected to the AcistCVi system (AcistCVi, Medical System Inc., Germany). The MX960 meets (or exceeds) the specifications (statements) of the Association for the Advancement of Medical Instrumentation/European Society of Hypertension/International Organization for Standardization (AAMI/ESH/ISO) Colla-boration. The AsistCVi system was synchronized with the X-ray imaging system Allura Xper FD10 or AlluraClarity FD20/10 (Philips Healthcare, the Netherlands).

Prior to each measurement, the combined system of catheter, tubing and external transducer was flushed with saline solution and the aoBP (or bBP) trace was visually inspected for quality. According to the calibration scheme recommended by the manu-facturer, the external pressure transducer was calibrated following the system’s inbuilt 2-point calibration method. First, the "zero" is assigned to the pressure value recorded when the sensor was opened to the atmosphere (adjusting the baseline to zero or at-mospheric pressure) and second by exposing the transducer to a pressure level equal to 100 mmHg (the device itself exposes the transducer to 100 mmHg, and the operator checks that is the pressure level displayed on the recording monitor). In the Cardiac Catheterization Laboratory of the Favaloro Foundation University Hospital, the dynamic response of the catheter, tubing, and external transducer combined system was adjusted to ensure a: (i) natural frequency of at least 20 Hz and (ii) damping coefficient of at least 0.3. It is to note that it was demonstrated that several external transducers, including the one used in the present work (MX960, LogiCal), have a high quality, distortion-free frequency response within the bandwidth of 0 to 30 Hz (Billiet, 2007). The quantitative limits described above guarantee an adequate compromise between the natural frequency and the damping coefficient, which ensures that measurement systems operate in areas of adequate dynamic responses or, at worst, in a very slightly under-damped region. The external transducer was maintained at heart (mid-axillary line) level. Invasive BP waveforms were visualized in the Allura Xper FD10 or AlluraClarity FD20/10 monitor images (Philips Healthcare, the Netherlands).

Simultaneously with invasively obtained aoBP recordings, aoBP estimates were obtained using three different approaches (vascular ultrasound, oscillometry/plethysmography and applanation tonometry) [see below] (Figure 1).

Once invasive aoBP recordings were obtained, the catheter was positioned in the contralateral BA at the level where the pneumatic cuff for non-invasive aoBP and bBP measurement (Mobil-O-Graph device) was located (Figure 1). Thereafter, intra-arterial bBP levels and waveforms were recorded and non-invasive bBP data were obtained (immediately before or after) using oscillometry (Mobil-O-Graph device) [see below]. Following each invasive bBP recording, the multipurpose 5 French catheter was again positioned in the ascending aorta and aoBP levels and waveforms were recorded, allowing hemodynamic stability to be assessed (confirmed).

Systolic, diastolic, mean (i.e., area under the pressure/time curve, divided by the cycle length) BP levels and HR were determined by means of invasive-derived data analysis processing systems. Once invasive and non-invasive measurement procedures concluded, the catheter was removed, and each subject returned to the recovery area. Finally, once the subject's clinical condition was considered stable, the patient was discharged from the University Hospital. No collateral harms or complications were observed during the invasive and non-invasive interventions.

1.3 Non-Invasive Measurement of Brachial Blood Pressure and Determination of Mean Arterial Blood Pressure

bBP pressure and waveforms were recorded by oscillometry/plethysmography (Mobil-O-Graph device), simultaneously and/or immediately before or after each invasive recording (at aortic or brachial level). Non-invasive bBP values were used to calibrate pulse waveforms recorded at CCA (vascular ultrasound), BA (applanation tonometry and oscillometry/plethysmography) and RA (applanation tonometry) level [see below].

The bSBP, bDBP, brachial pulse pressure (bPP, bPP=bSBP-bDBP), and HR values obtained with the oscillometric system (Mobil-O-Graph device) were named ´bSBPosc´, ´bDBPosc´, ´bPPosc´ and ´HRosc´, respectively. In turn, the bMBP directly obtained with oscillometry (the point of lower bBP for maximal oscillations) was identified as ´bMBPosc´. Additionally, using the bSBPosc and bDBPosc, bMBP was quantified (calculated) as (Papaioannou et al., 2016-B; Agnoletti et al., 2012; Chemla et al., 2005):

(i) $bMBP_{0.33}$ [mmHg] = bDBPosc+0.33*bPPosc

(ii) $bMBP_{0.33HR}$ [mmHg] = bDBPosc+[0.33+(0.0012*HRosc)]*bPPosc

(iii) $bMBP_{0.412}$ [mmHg] = bDBPosc+0.412*bPPosc

As described, the approaches use two different "form factors", defined as the percentage of the waveform amplitude (in other words the bPP) that is added to the minimum (the bDBPosc) to obtain the mean value (the bMBP) (Mahieu et al. 2010). These different ways of obtaining bMBP were subsequently used to calibrate the ultrasound, tonometry and oscillometry/plethysmography recordings used to obtain aoBP, with the aim of determining whether any of these equations allow for smaller differences between aoSBP (and aoPP) obtained invasively and non-invasively.

1.4 Non-Invasive Measurement of Central Aortic Blood Pressure

Simultaneously with invasive aoBP recordings, aoBP estimates were obtained (random order) using different non-invasive approaches, whose recording techniques and mathematical algorithms are detailed below.

1.4.1 aoBP estimated from brachial oscillometry/plethysmography recordings

Oscillometric/plethysmographic bBP levels and waveforms recordings were obtained with the Mobil-O-Graph automatic device (Model PWA, IEM GmbH, Stolberg, Germany). To this end, a pneumatic cuff properly sized according to the patient characteristics (Zinoveev et al., 2019; Zócalo and Bia, 2022; García-Espinosa et al., 2016) was positioned in the arm (in our case in the contralateral to the used for the sheath insertion) (Figure 1) (Weber et al., 2011). Then, HRosc and bMBPosc were registered, and bSBPosc and bDBPosc were obtained, by means of internal algorithms of the device manufacturer. In turn, aoBP levels and waveforms were estimated from BA recordings using a validated GTF. Only high-quality records (index equal to 1 or 2) and satisfactory waveforms (visual inspection) were considered.

The Mobil-O-Graph device determines bBP and aoBP during the same 'double' inflation-deflation cycle of the cuff. Taking into account this, the device was used: (i) to determine aoBP, and (ii) to determine the bSBPosc, bMBPosc and bDBPosc values used in its own calibration and in the calibration of other approaches. Therefore, every time bBP was measured with this device, along with the measurement with another non-invasive approach (e.g. vascular ultrasound), aoBP was also determined using the Mobil-O-Graph.

Each aoBP data derived from Mobil-O-Graph recordings was obtained calibrating to: (i) bSBPosc and bDBPosc (SD approach); (ii) bDBPosc and bMBPosc and (iii) bDBPosc and bMBP$_{0.33.}$ It was not possible to calibrate Mobil-O-Graph derived data using invasive bBP levels (catheterism-derived) or other forms of estimating bMBP (i.e., bMBP$_{0.33HR}$ and bMBP$_{0.412}$) or), as the device does not allow it.

1.4.2 aoBP estimated from carotid ultrasonography recordings

Left CCAs were visualized a centimeter proximal to the bulb using ultrasound (6-13 MHz, M-Turbo, Sonosite Inc., Bothell, WA, USA). Sequences of images (30 s, B-Mode, longitudinal views) were stored for off-line analysis in which beat-to-beat diameter waveforms were obtained using border detection software (Hemoydin4M software, Dinap s.r.l., Buenos Aires, Argentina). Then, aoBP waveform and values were obtained from the diameter data (Zócalo et al. 2013; Van Bortel et al., 2001; Vermeersch et al., 2008). To this end, as in previous works (Zócalo et al., 2013), CCA diameter waveforms were calibrated using an exponential calibration scheme, applying the method proposed by Vermeersch et al. that assumes an exponential pressure-diameter relationship: $\boldsymbol{p(t) = p_d exp\left[\alpha\left(\frac{A(t)}{Ad} - 1\right)\right]}$, **with** $\boldsymbol{A(t) = \frac{\pi d^2(t)}{4}}$ **, and** $\boldsymbol{\alpha = \frac{Ad\,(In\frac{SBP}{DBP})}{As-Ad}}$**,** where p(t) is pressure, d(t) is diameter, A(t) is arterial cross-section as a function of time, DBP and SBP are end-diastolic and peak systolic BP, respectively, Ad and As are end-diastolic and peak systolic cross-section area, respectively, and α is the BP-independent wall stiffness coefficient (Vermeersch et al., 2008). To use this equation to calculate the BP waveform from a given diameter waveform, systolic and diastolic pressures must be known at the same site as the arterial cross section. Assuming that (in supine position) DBP and MBP remain constant throughout large arteries, the iterative scheme can be used to determine α based on bMBP and bDBP. To this end, (i) invasive-derived bDBP and bMBP, and (ii) bDBPosc and bMBP levels were used to calibrate CCA diameter waveforms. Specifically, CCA ultrasound-derived aoSBP and aoPP were obtained using four different calibration schemes that included bDBPosc in conjunction with: (i) bMBPosc, and bMBPcalc [(ii) bMBP$_{0.33}$, (iii) bMBP$_{0.33HR}$, and (iv) bMBP$_{0.412}$].

1.4.3 aoBP estimated from carotid applanation tonometry recordings

Central aoBP levels and waveforms were obtained (random order) using applanation tonometry (SphygmoCor-CvMS ([SCOR]; v.9, AtCor-Medical, Sydney, NSW, Australia) applied to CCA, RA, and BA (Figure 1). Applanation tonometry provides the beat-to-beat BP waveform signal (10 seconds) that can be calibrated using different calibration schemes. Only accurate waveforms on visual inspection and high-quality recordings (in-device quality control (operator) index > 75%) were considered. About this, the operator index indicates the overall reproducibility of the signals. It is calculated by assessing weighted quality control parameters and adding them to give a number as a percentage. The index considers: (i) average height of individual recordings, (ii) pulse height variation (accepted: <5%), (iii) diastolic variation (indicates constancy of the basal level, accepted: <5%), (iv) shape variation (accepted: <5%), and (v) maximum dP/dt (maximum value of the first derivative or maximal rate of wave rise).

From the CCA tonometry-derived recordings, aoBP levels were obtained: (i) applying a carotid-aortic GTF (´GTF approach´) and (ii) without using a GTF (not-processed or ´NPROC´ approach´), considering CCA and ascending aorta waveforms to be identical due to the proximity of the arterial sites (Karamanoglu and Feneley, 1996; Chi et al., 2022).

Disregard of the approach considered to obtain aoBP levels from CCA tonometry-derived data (as well as from RA and BA data as described below) signals were calibrated to: (i) invasively-derived bDBP and bMBP, and non-invasively derived, (ii) bSBPosc and bDBPosc, and (iii) bDBPosc and bMBP, using different ways to quantify bMBP: bMBPosc, $bMBP_{0.33}$, $bMBP_{0.33HR}$, $bMBP_{0.412}$.

1.4.4 aoBP estimated from radial applanation tonometry recordings

Central aoBP levels and/or waveforms were obtained from RA tonometry-derived pulse waveforms, considering four different data analysis approaches.

First, the aoBP waveform, aoSBP and aoPP levels were obtained applying a radial-to-aortic GTF (manufacturer´s property) (Zinoveev et al., 2019; Zócalo and Bia, 2022).

Second, aoSBP and aoPP were quantified from the second peak of the RA pulse waveform (usually referred to as 'second shoulder, P2 or SBP2´) (Pauca et al., 2004). About this, ascending aorta BP waveform has an early peak (S1), attributed to the forward traveling wave and a late higher systolic peak (S2), due to the augmentation of the systolic phase by the reflected pressure wave component. Conversely, at the level of the RA there is an early high peak (S1), representing the forward traveling wave, and a shorter second peak (S2 or SBP2) due to the 'delayed' arrival of the reflected wave. It has been also proposed that the SBP2 of the RA waveform represents aoSBP and thus could serve as its surrogate marker (Pauca et al., 2004). This 'unexpected' observation has been reproduced in non-invasive and invasive studies, which verified that, in general, the SBP2 of the RA waveform is an accurate estimate of the aoSBP (Protogerou et al., 2010; Hickson et al., 2009).

Third, aoSBP and aoPP were quantified applying a first-order low-pass filter. Each single point in the recorded signal (e.g., recorded RA waveform) is summed up with its neighbors and the result is divided by the number of points considered. The more data points are taken into the average formula, the smoother the signal. The method is entitled ´N-point moving average´ (NPMA) (Williams et al., 2011). Because of its filtering characteristics, the NPMA method provides aoBP systolic levels (enabling obtaining aoPP), but in contrast to other methods (e.g., radial-to-aortic GTF), no information about aoBP waveforms is given (Weber et al., 2014). NPMA was initially proposed as a

simple method to estimate aoSBP from RA-derived BP waveforms (Williams et al., 2011). Then it was proposed it could be also used to analyze BA-derived BP waves (Shih et al., 2014). The number of averaged points (´N´) differs depending on the pressure waves (RA or BA) considered. For instance, N=Fs/4 (Williams et al. 2011) or N=Fs/4.4 (Xiao et al., 2018) for RA-derived waves, and N=Fs/6 for waves obtained from BA recordings (Shih et al., 2014) (Fs is the sampling frequency, and the numbers 4.0, 4.4 and 6 represent the optimal integer denominator ´K´). Recently Xiao et al. reported that K=4.4 would estimate aoSBP more accurately than K=4.0 (Xiao et al. 2018). With this in mind, in this work we applied the NPMA method using both denominators (4.0 and 4.4) when analyzing RA-tonometry recordings. Using the SphygmoCor device (Fs=128 Hz), 32 (128/4) and 29 (128/4.4) points were averaged for RA waves, while 21 (128/6) points were considered when analyzing BA records [see below].

1.4.5 aoBP estimated from brachial applanation tonometry recordings

From tonometry-derived BA pulse waveforms (calibrated using different methods), we quantified aoBP: (i) applying the SphygmoCor GTF, and (ii) using NPMA method (k=6) (Shih et al. 2014).

1.5 Carotid, Radial and Brachial Artery Waveforms Calibration

Virtually all devices that allow non-invasive acquisition of the pulse waveforms require a calibration step. In example, waveforms recorded with tonometry or vascular ultrasound require 'pressure calibration' to transform, respectively, voltage (e.g., mV) or diameter (e.g., mm) signals into pressure (e.g., mmHg) waves. Different approaches were considered to calibrate the peripheral waveform:

1. Invasive-derived (´Inv´): bMBP and bDBP invasively obtained (BA catheterization) were respectively assigned, to the algebraic mean and minimum of the CCA, RA or BA waveforms non-invasively recorded.

2. Systo-diastolic (´SD´): bSBPosc and bDBPosc were respectively assigned to the maximum and minimum of the CCA, BA or RA waveforms non-invasively recorded.

3. Oscillometric-derived (´Osc´): bMBPosc and bDBPosc were respectively assigned to the algebraic mean and minimum of the CCA, RA or BA waveforms non-invasively recorded.

4. Calculated MBP: calculated bMBP ($bMBP_{033}$, $bMBP_{033HR}$ and $bMBP_{0412}$) and bDBPosc were respectively assigned to the algebraic mean and minimum of the CCA, RA or BA waveforms non-invasively recorded.

1.6 Variable names: aoSBP and aoPP

In order to systematize their definition and to facilitate their identification throughout the text, tables and figures, the variables were named as a sequence of terms (separated by underscores) referring to (Figure 1):

(i) the biological variable analyzed: aoSBP or aoPP

(ii) the recording technique/device used: ´MOG´ (Mobil-O-Graph: oscillometry/plethysmography); ´SCOR´ (SphygmoCor: applanation tonometry); ´Echo´ (Echography or vascular ultrasound),

(iii) the arterial site of recording: ´CT´ or ´CCA´ (carotid tonometry or carotid ultrasound, respectively), ´BT´ (brachial artery tonometry), ´RT´ (radial artery tonometry),

(iv) the analysis considered: ´GTF´ (use of a general transfer function), ´NPMA´ (use of the N-point moving average filter; indicating the filtering factor used in RA: ´4.0´ or ´4.4´, and in BA: ´6.0´), ´ExpAdj´ (use of an exponential fit for the diameter-pressure transformation),

(v) the calibration form of the recorded waves: ´Inv´, ´sd´, ´osc´, or the specific equation (form factor) used to calibrate using the calculated bMBP (´033´, ´033HR´, ´0412´).

As an example, the variable "aoSBP (MOG_GTF_sd)" refers to the aortic systolic blood pressure (´aoSBP´) measured with the Mobil-O-Graph (´MOG´), using a generalized transfer function (´GTF´) and obtained when calibrating using the systo-diastolic scheme (´sd´).

1.7 Data and Statistical Analysis

Invasive and non-invasive bBP and aoBP data obtained with the different techniques, recording sites, data analyses and/or calibration schemes are shown in the Supplementary File 2, Tables S1-S5. Tables S1 to S5 show: (i) aortic and brachial data invasively obtained, (ii) bBP values used to calibrate the signals (obtained simultaneously with the Mobil-O-Graph) and (iii) aoBP data obtained from the non-invasive approaches: Table S1: Oscilometry/Plethismography (Mobil-O-Graph), Table S2: RA applanation tonometry (SCOR), Table S3: CCA applanation tonometry (SCOR), Table S4: BA applanation tonometry (SCOR), Table S5: CCA ultrasound (SonoSite + Hemoydin 4M software).

After analyzing the subjects´ characteristics, aoBP and bBP data obtained with the different approaches (Tables 1 and 2; Supplementary File 2, Table S1-S5), we analyzed the association and agreement between invasive and non-invasive aoSBP and aoPP data. To this end, Lin’s Concordance Correlation Coefficient (CCC) (Figure 2; Supplementary File 2, Table S6 (for aoSBP) and Table S7 (for aoPP)), and Bland-Altman analysis were considered (Supplementary File 2, Table S8 and Table S9). Bland-Altman analysis enabled to determine mean (systematic) and proportional errors (bias) between aoSBP (Figure 3 and 4) and aoPP (Figure 5 and 6) data obtained with the reference (invasive) method and the non-invasive ones.

Figure 7 and Figure 8 (for aoSBP), and Figure 9 and 10 (for aoPP) show the pooled results (errors derived from the Bland-Altman analysis), when considering: (i) the recording and analysis methodology (regardless of calibration scheme), (ii) the calibration scheme (regardless of the recording and analysis methodology), and (iii) the recording site (CCA, BA or RA).

In all cases, Bland-Altman analysis correspond to the reference method (invasive aoSBP or aoPP data; x-axis) against the non-invasive and invasive difference (non-invasive minus invasive data; y-axis). The corresponding linear regression equations were obtained. Systematic error was considered present if mean error was significantly different from zero; proportional error was considered present if the slope of the linear regression was statistically significant. Considering the mean and proportional errors (regression equation) obtained from Bland-Altman analysis, the mean difference between (i) invasive and (ii) non-invasive derived aoSBP and aoPP, was calculated (and graphed) for different aoSBP (Figure 4-D and Figure 7-B) and aoPP (Figure 6-D and Figure 9-B) values.

According to the central limit theorem, taking into account Kurtosis and Skewness coefficients distribution and the number of subjects (sample size>25) a normal distribution was considered

(Lumley et al., 2002). Data analyses were done using MedCalc (v.14.8.1, MedCalc Inc., Ostend, Belgium) and IBM-SPSS Statistical Software (v.26, SPSS Inc., Illinois, USA). A $p<0.05$ was considered statistically significant.

2 References

Agnoletti, D., Zhang, Y., Salvi, P., Borghi, C., Topouchian, J., Safa,r M.E., et al. (2012). Pulse pressure amplification, pressure waveform calibration and clinical applications. *Atherosclerosis*. 224(1):108-12. doi: 10.1016/j.atherosclerosis.2012.06.055.

Bia, D., Zócalo, Y. (2021). Physiological age- and sex-related profiles for local (aortic) and regional (carotid-femoral, carotid-radial) pulse wave velocity and center-to-periphery stiffness gradient, with and without blood pressure adjustments: reference intervals and greement between methods in healthy subjects (3-84 Years). *J Cardiovasc Dev Dis*. 8(1):3. doi: 10.3390/jcdd8010003.

Billiet, E. (2007). Tested disposable invasive blood pressure transducers all perform excellently. *ICU Managment* 7(2):12-15

Chemla, D., Antony, I., Zamani, K., Nitenberg, A. (2005). Mean aortic pressure is the geometric mean of systolic and diastolic aortic pressure in resting humans. *J Appl Physiol (1985)*. 99(6):2278-2284. doi:10.1152/japplphysiol.00713.2005

Chi, C., Lu, Y., Zhou, Y., Li, J., Xu, Y., Zhang, Y. (2022). Factors that may impact the noninvasive measurement of central blood pressure compared to invasive measurement: The MATCHY Study. *J Pers Med.* 12(9):1482.doi:10.3390/jpm12091482

García-Espinosa, V., Curcio, S., Marotta, M., Castro, J.M., Arana, M., Peluso, G., et al. (2016). Changes in central aortic pressure levels, wave components and determinants associated with high peripheral blood pressure states in childhood: analysis of hypertensive phenotype. *Pediatr Cardiol.* 37(7):1340-1350. doi:10.1007/s00246-016-1440-5

Hickson, S.S., Butlin, M., Mir, F.A., Graggaber, J., Cheriyan, J., Khan, F., et al. (2009). The accuracy of central SBP determined from the second systolic peak of the peripheral pressure waveform. *J Hypertension*, 27(9), 1784–1788. https://doi.org/10.1097/hjh.0b013e32832e0b58

Karamanoglu, M., Feneley, M.P. (1996). Derivation of the ascending aortic-carotid pressure transfer function with an arterial model. *Am J Physiol.* 271(6Pt2):H2399-H2404. doi:10.1152/ajpheart.1996.271.6.H2399

Lumley, T., Diehr, P., Emerson, S., Chen, L. (2002). The importance of the normality assumption in large public health data sets. *Annu Rev Public Health* 23:151–69. doi: 10.1146/annurev.publhealth.23.100901.140546.

Mahieu, D., Kips, J., Rietzschel, E.R., De Buyzere, M.L., Verbeke, F., Gillebert, T.C., et al. (2010). Noninvasive assessment of central and peripheral arterial pressure (waveforms): implications of calibration methods. J Hypertens. 28(2):300-305. doi:10.1097/HJH.0b013e3283340a1a

Papaioannou, T.G., Protogerou, A.D., Vrachatis, D., Konstantonis, G., Aissopou, E., Argyris, A., et al. (2016). Mean arterial pressure values calculated using seven different methods and their associations with target organ deterioration in a single-center study of 1878 individuals. *Hypertens Res.* 39(9):640-7. doi: 10.1038/hr.2016.41. (B)

Pauca, A.L., Kon, N.D., O'Rourke, M.F. (2004). The second peak of the radial artery pressure wave represents aortic systolic pressure in hypertensive and elderly patients. *British Journal of Anaesthesia*, 92(5), 651–657. https://doi.org/10.1093/bja/aeh121

Protogerou, A.D., Nasothimiou, E.G., Papadogiannis, D. (2010). The second systolic radial blood pressure peak predicts cardiovascular risk only in subjects below 50 years of age. *Hypertens Res* 33(4), 289–290. https://doi.org/10.1038/hr.2010.14

Sánchez, R., Pessana, F., Lev, G., Mirada, M., Mendiz, O., Ramírez, A., et al. (2020). Central blood pressure waves assessment: a validation study of non-invasive aortic pressure measurement in human beings. *High Blood Press Cardiovasc Prev.* 27(2):165-174. doi: 10.1007/s40292-020-00371-4.

Shih, Y.T., Cheng, H.M., Sung, S.H., Hu, W.C., Chen, C.H. (2014). Application of the N-point moving average method for brachial pressure waveform-derived estimation of central aortic systolic pressure. *Hypertension.* 63(4):865-870. doi:10.1161/HYPERTENSIONAHA.113.02229

Van Bortel, L.M., Balkestein, E.J., van der Heijden-Spek, J.J., Vanmolkot, F.H., Staessen, J.A., Kragten, J.A., et al. (2001). Non-invasive assessment of local arterial pulse pressure: comparison of applanation tonometry and echo-tracking. *J Hypertens* 19(6), 1037–1044. https://doi.org/10.1097/00004872-200106000-00007

Vermeersch, S.J., Rietzschel, E.R., De Buyzere, M. L., De Bacquer, D., De Backer, G., Van Bortel, L.M., et al. (2008). Determining carotid artery pressure from scaled diameter waveforms: comparison and validation of calibration techniques in 2026 subjects. *Physiol Meas* 29(11), 1267–1280. https://doi.org/10.1088/0967-3334/29/11/003.

Weber, T., Wassertheurer, S., Rammer, M., Maurer, E., Hametner, B., Mayer, C.C., et al. (2011). Validation of a brachial cuff-based method for estimating central systolic blood pressure. *Hypertension.* 2011;58(5):825-832. doi:10.1161/HYPERTENSIONAHA.111.176313

Weber, T., Wassertheurer, S. (2014). Moving on-on average in the right direction?: noninvasive methods to estimate central blood pressure. *Hypertension.* 63(4):665-667. doi:10.1161/HYPERTENSIONAHA.113.02885

Williams, B., Lacy, P.S., Yan, P., Hwee, C.N., Liang, C., Ting, C.M. (2011). Development and validation of a novel method to derive central aortic systolic pressure from the radial pressure

waveform using an n-point moving average method. *J Am Coll Cardiol.* 57(8):951-961. doi:10.1016/j.jacc.2010.09.054

Xiao, H., Butlin, M., Qasem, A., Tan, I., Li, D., Avolio, A.P. (2018). N-Point moving average: a special generalized transfer function method for estimation of central aortic blood pressure. *IEEE Trans Biomed Eng.* 65(6):1226-1234. doi:10.1109/TBME.2017.2710622

Zinoveev, A., Castro, J.M., García-Espinosa, V., Marin, M., Chiesa, P., Bia, D., et al. (2019). Aortic pressure and forward and backward wave components in children, adolescents and young-adults: agreement between brachial oscillometry, radial and carotid tonometry data and analysis of factors associated with their differences. *PLoS One.* 14(12):e0226709. doi: 10.1371/journal.pone.0226709.

Zócalo, Y., Bia, D. (2021). Sex- and age-related physiological profiles for brachial, vertebral, carotid, and femoral arteries blood flow velocity parameters during growth and aging (4-76 years): comparison with clinical cut-off levels. *Front Physiol.* 12:729309. doi: 10.3389/fphys.2021.729309. (A)

Zócalo, Y., Bia, D. (2021). Age- and sex-related profiles for macro, macro/micro and microvascular reactivity indexes: Association between indexes and normative data from 2609 healthy subjects (3-85 years). *PLoS One.* 16(7):e0254869. doi: 10.1371/journal.pone.0254869.(B)

Zócalo, Y., Bia, D. (2022). Central pressure waveform-derived indexes obtained from carotid and radial tonometry and brachial oscillometry in healthy subjects (2-84 y): age-, height-, and sex-related profiles and analysis of indexes agreement. *Front Physiol.* 12:774390. doi: 10.3389/fphys.2021.774390.

Zócalo, Y., Bia, D., Armentano, R.L., González-Moreno, J., Varela, G., Calleriza, F., et al. (2013). Resynchronization improves heart-arterial coupling reducing arterial load determinants. *Europace*. 15(4):554-565. doi:10.1093/europace/eus285

Zócalo, Y., García-Espinosa, V., Castro, J.M., Zinoveev, A., Marin, M., Chiesa, P., et al. (2021). Stroke volume and cardiac output non-invasive monitoring based on brachial oscillometry-derived pulse contour analysis: Explanatory variables and reference intervals throughout life (3-88 years). *Cardiol J*. 6:864-878. doi: 10.5603/CJ.a2020.0031.
